# Shrub expansion raises both aboveground and underground multifunctionality on a subtropical plateau grassland: coupling multitrophic community assembly to multifunctionality and functional trade-off

**DOI:** 10.3389/fmicb.2023.1339125

**Published:** 2024-01-11

**Authors:** Leilei Ding, Hong Chen, Mengya Wang, Puchang Wang

**Affiliations:** ^1^Guizhou Institution of Prataculture, Guizhou Academy of Agricultural Sciences, Guiyang, Guizhou, China; ^2^Guizhou Songbaishan Reservoir Management Office, Guiyang, Guizhou, China; ^3^College of Animal Science, Guizhou University, Guiyang, Guizhou, China; ^4^School of Life Science, Guizhou Normal University, Guiyang, Guizhou, China

**Keywords:** shrub expansion, aboveground and underground multifunctionality, multitrophic community assembly, functional trade-off, abundant and rare taxa, archaea, nematodes, protists

## Abstract

**Introduction:**

Shrubs have expanded into grasslands globally. However, the relative importance of aboveground and underground diversity and the relative importance of underground community assembly and diversity in shaping multifunctionality and functional trade-offs over shrub expansion remains unknown.

**Methods:**

In this study, aboveground and underground multitrophic communities (abundant and rare archaea, bacteria, fungi, nematodes, and protists) and 208 aboveground and underground ecosystem properties or indicators were measured at three stages (Grass, Mosaic, Shrub) of shrub expansion on the Guizhou subtropical plateau grassland to study multifunctionality and functional trade-offs.

**Results:**

The results showed that shrub expansion significantly enhanced aboveground, underground, and entire ecosystem multifunctionality. The functional trade-off intensities of the aboveground, underground, and entire ecosystems showed significant V-shaped changes with shrub expansion. Shrub expansion improved plant species richness and changed the assembly process and species richness of soil abundant and rare subcommunities. Plant species diversity had a greater impact on multifunctionality than soil microbial diversity by more than 16%. The effect of plant species diversity on functional trade-offs was only one-fifth of the effect of soil microbial diversity. The soil microbial species richness did not affect multifunctionality, however, the assembly process of soil microbial communities did. Rather than the assembly process of soil microbial communities, the soil microbial species richness affected functional trade-offs.

**Discussion:**

Our study is the first to couple multitrophic community assemblies to multifunctionality and functional trade-offs. Our results would boost the understanding of the role of aboveground and underground diversity in multifunctionality and functional trade-offs.

## Introduction

Grasslands cover 54% of the terrestrial land area (Barber-Cross et al., 2022) and 69% of the agricultural land area (Song and Wang, 2022). They provide over $1,000 billion in ecosystem services to 36% of the global human population (Liao et al., 2018). However, shrub expansion (Ding et al., 2020), a top driver of biome and land cover transitions (Loss et al., 2021) defined as an increase in the cover, density, or biomass of shrub or woody plant species (Eldridge and Ding, 2020; Ambrosino et al., 2023), is occurring on Dengzeng et al. (2022) one-tenth to one-fifth of the global grasslands (Idbella et al., 2022). Shrub expansion has affected over 500 million hectares of grassland (Ding and Eldridge, 2023) and over two billion people (Qu et al., 2023). Furthermore, the global area of shrub expansion is predicted to increase in the coming years (Chandregowda et al., 2018; Idbella et al., 2022; Ding and Eldridge, 2023).

Shrub expansion triggers major ecosystem changes (Cannone et al., 2022) with positive and negative effects on aboveground vegetation (Drees et al., 2023) and belowground soil ecosystems (Yannarell et al., 2014; Ding et al., 2020; Ma et al., 2023). However, it remains debatable whether shrub expansion improves or degrades land. Some studies have shown that shrub expansion should be regarded as a degradation process (Khazieva et al., 2022; Root-Bernstein and Hoag, 2022; Qu et al., 2023) due to its generally negative effects on biodiversity (Ding et al., 2020; Guo et al., 2022; Song and Wang, 2022; Gómez-García et al., 2023), livestock production (Eldridge and Ding, 2020; Root-Bernstein and Hoag, 2022), economy (Drees et al., 2023), and ecosystem functions (Li et al., 2022b). Other studies have revealed that shrubs do not have a negative effect on soil quality (Ambrosino et al., 2023) but enhance nutrient cycling, primary production, soil water and nutrients (Chandregowda et al., 2018; Li D. et al., 2023), and bacterial diversity (Guo et al., 2022). Although the impact of shrub expansion on ecological processes and functioning has been a subject of growing interest (Liu Y. F. et al., 2023), most studies have focused on single functions (Idbella et al., 2022). This hinders an integrated understanding of the overall functioning of shrub expansion ecosystems.

Ecosystem multifunctionality refers to the ability of ecosystems to simultaneously deliver multiple functions (Garland et al., 2020). Multifunctionality can provide a broad and integrative insight into (Delgado-Baquerizo et al., 2020) ecological patterns and ecosystem functioning (Manning et al., 2018) by commonly synthesizing community-level properties and/or processes (Luo et al., 2018; Manning et al., 2018) on a small-scale to even microscale (Hölting et al., 2019; Ding and Wang, 2021). Despite the upsurging interest in multifunctionality (Slade et al., 2017, Xu et al., 2022), multifunctionality changes in response to shrub expansion remain poorly understood (Chandregowda et al., 2018). Although the relationship between biodiversity and multifunctionality has been extensively explored (Wagg et al., 2014; Zhang et al., 2023; Wang et al., 2023b), little is known about the extent to which shrub expansion drives the multifunctionality of aboveground, underground, and entire ecosystems. Although the effects of shrub expansion on aboveground and belowground communities have been studied extensively (Eldridge et al., 2011; Sepp et al., 2021; Dengzeng et al., 2022; Guo et al., 2022; Ding and Eldridge, 2023; Wang et al., 2023b), the relative contributions of aboveground (plant community) and belowground (soil microbial community) diversity to multifunctionality remain largely unresolved (Berlinches et al., 2022; Wang et al., 2023b), especially for shrub expansion succession.

Mounting evidence supports the idea that soil microbial diversity significantly affects various ecosystem functions, both individually and simultaneously (Sokol et al., 2022; Ding et al., 2023). However, many studies have ignored the role of community assembly processes in shaping both biodiversity and ecosystem functions consequently (Ding and Wang, 2021). Revealing community assembly and associated influencing factors is a long-standing (Hao et al., 2021), extremely challenging (Luan et al., 2020), and essential (Luan et al., 2020) effort for understanding diversity, ecological properties, and functioning (Cao et al., 2022; Zhang et al., 2022).

Nevertheless, the mechanisms by which regional, local, abundant, and rare taxa assemble along with shrub expansion remain unknown (Qu et al., 2023). Although it is increasingly recognized that microbial community assembly determines microbial diversity during ecological succession, affecting ecosystem functioning (Cao et al., 2022), the cascading linkages from assembly to microbial diversity, and in turn to multifunctionality, remain to be established. As these processes determine how the effect of diversity occurs (Leibold et al., 2017), we speculate that the role of the assembly process may outweigh that of diversity.

Recent studies have begun to demonstrate the changes in the trade-offs among functions (Wang et al., 2022b). Trade-off is defined as a state in which one ecosystem function increases while another decreases (Lu et al., 2014) in a narrow sense. However, in a broader sense, trade-offs refer to unidirectional changes with uneven paces (Lu et al., 2014) in ecosystem functions. Although this trade-off is common among ecosystem functions (Felipe-Lucia et al., 2018), changes in the intensity of ecosystem functional trade-offs during shrub expansion and the driving forces behind these changes are still poorly investigated. A full understanding of the trade-off among functions is needed for better management of shrubs (Nerlekar et al., 2022).

In this study, 208 aboveground and underground ecosystem properties or indicators were used to quantify the multifunctionality and functional trade-offs of aboveground, underground, and entire ecosystems, and soil multitrophic communities (archaea, bacteria, fungi, nematodes, and protists) were assessed to test the following hypotheses: (1) shrub expansion can reshape the microbial community assembly process of soils (H1) and (2) the impact of microbial community assembly processes is greater than the impact of microbial diversity in shaping multifunctionality (H2) and functional trade-off intensity (H3). This study would provide novel insights into the biotic driving forces that reshape ecosystem functioning and functional trade-offs in subtropical grasslands following shrub expansions.

## Materials and methods

### Study area, experimental design, and sampling

The study was conducted in a 60 km^2^ natural grassland (E107°4′ − 11′, N26°9′ − 19′, 1,500–1,700 m a. s. l.) in Longli county on the Guizhou plateau, southwestern China. The region has a humid subtropical monsoon climate. The annual average air temperature is about 14.7°C. The average temperature of the coldest month is 4.6°C, and the average air temperature of the hottest month is 23.6°C (Ding et al., 2023). The annual sunshine hours are nearly 1,160 h, and the number of non-frost periods is 283 days (Ding et al., 2023). The average annual precipitation is 1,160 mm, which falls mainly between April and October (Ding et al., 2020). The regional soil type is Haplic alisols, and the main species in the grassland are *Eulalia pallens*, *Arundinella hirta*, and *Carex cruciata wahlenb* (Ding et al., 2020, 2023).

Shrub expansion on open grasslands formed operational experiments in a natural and realistic environment, where the grass, mosaic (transition), and shrub stages coexisted in nearly identical topography, climate, and soil type. This natural scenario was instrumental in uncovering the impact of shrub expansion on vegetation and soil microbiomes (Sepp et al., 2021). Based on the process of shrub expansion succession and the ecological characteristics of native species (Ding et al., 2019, 2020), we selected 15 sites representing the three stages (grass, mosaic, and shrub) with five spatially independent replicates from November to December 2021. At each site, a 1 m × 1 m plot was established for sampling. After plant species richness (number of plant species), plant cover, and shrub cover in a plot were recorded, the aboveground stand biomass of the vegetation was cut by leveling the ground, and all litter covering the ground was collected. These vegetation samples were killed at 105°C, dried to constant weight at 65°C, and then weighed. Sixty-one soil cores (5 cm diameter × 5 cm deep) uniformly distributed in the plot were drilled using ring knives (50-mm inner diameter × 50-mm height). Fifty soil cores were mixed. These mixed samples were then divided into several parts. One part was quickly packed into sterile self-sealing bags and stored at −86°C until the determination of microorganisms. To avoid cross-contamination (Wang et al., 2022a), separate ring knives and medical sterile gloves were used. One part was used to measure soil physical and chemical properties, and the other part was used to measure soil enzyme activities. The roots in five soil cores were collected and killed at 105°C after assessing the root morphology (WinRHIZO Pro2016, REGENT, Canada) They were dried to constant weight at 65°C and then weighed. The roots in another five soil cores were collected, and the washing solution of these roots was used to determinate root-related organic acids and acidic phosphate activity. One soil core was used to determine the bulk density, soil porosity, and capillary porosity. The assay methods are described in the Supplementary document. This study focused on the topsoil because many previous studies have focused on the topsoil (Allison et al., 2015; Ding et al., 2020; Kakeh et al., 2022). Moreover, a recent study found that shrub expansion altered the microbial community in the topsoil rather than in the subsoil (Droma et al., 2023).

### Individual functions

A total of 208 functional variables were included in this study. The aboveground functions were characterized as follows: plant reservoir functions (plant species richness), aboveground productivity (Jing et al., 2021; Wang et al., 2023a) [aboveground stand biomass, litter, and total aboveground biomass = aboveground stand biomass + litter], and erosion regulation (Garland et al., 2020) [plant cover, shrub cover, and non-shrub cover = plant cover – shrub cover]. The underground functions were characterized as follows: nutrient provisioning (Jiao et al., 2022a) [microbial biomass nitrogen (MBN, mg/kg), microbial biomass phosphorus (MBP, mg/kg), total nitrogen (TN, g/kg), total potassium (TK, g/kg), total phosphorus (TP, g/kg), available nitrogen (AN, g/kg), available phosphorus (AP, mg/kg), and available potassium (AK, g/kg)], element cycling (Jiao et al., 2022a) [twenty soil carbon indicators; three molar stoichiometric ratios of soil carbon, nitrogen, and phosphorus; microbial carbon limitation (C_limitation); microbial nitrogen limitation (N_limitation); exchangeable calcium (E_Ca, cmol (1/2 Ca2+)/kg); exchangeable magnesium (E_Mg, cmol (1/2 Ca2+)/kg); dithionite-extractable Fe (D_Fe2O3, g/kg); dithionite-extractable Al (D_Al2O3, g/kg); organically complexed Fe oxides (OC_Fe2O3, g/kg); organically complexed Al oxides (OC_Al2O3, g/kg); poorly crystalline Fe oxyhydroxides (PC_Fe2O3, g/kg); poorly crystalline Al oxyhydroxides (C_Al2O3, g/kg); root organic acid; root acid phosphatase; forty plant residue indicators; forty-eight enzyme activities related to soil carbon, nitrogen, and phosphorus cycling (Luo et al., 2018, Wang et al., 2021); and twenty microbial residue indicators], functions related to plant health (Delgado-Baquerizo et al., 2020; Jiao et al., 2022a,b; Fan et al., 2023) [functions related to soil-borne plant pathogen control (reduced relative abundance of fungal plant pathogen and reduced relative abundance of pathotroph fungi), nutrient acquisition (relative abundance of arbuscular mycorrhizal fungi, relative abundance of ectomycorrhizal fungi, relative abundance of symbiotrophic fungi, eight indicators of fungal infection of roots, and seven root morphology indicators)], water regulation (Wang J. et al., 2022) [water content (WC, %), total porosity (Totalporosity, %), capillary porosity (CP, %), and non-capillary porosity (NCP, %)], soil physical context functions (Ding and Wang, 2021; Ding et al., 2023) [pH, bulk density (BD, g/cm^3^)], underground productivity [root biomass (g)], and antibiotic resistance gene control (Delgado-Baquerizo et al., 2020; Fan et al., 2023) [determined as −1 × relative abundance of twenty-two antibiotic resistance genes].

The methods of determination and calculation were described in our previous research (Ding et al., 2020) and in the Supplementary document. These individual functions were included in the multifunctionality because they are either real functions or good markers of ecosystem functions and have been frequently used to quantify ecosystem multifunctionality in recent studies (Manning et al., 2018; Ding and Wang, 2021; Guan et al., 2022; Kakeh et al., 2022; Ding et al., 2023; Jia et al., 2023; Luo et al., 2023; Yang et al., 2023a).

### Sequencing of soil gene amplicons and processing of sequencing data

To analyze soil archaea, bacterial, fungal, nematode, and protist communities, the total genomic DNA was extracted from soil samples using MOBIO Power Soil® DNA Isolation Kit (MOBIO Laboratories, Carlsbad, CA, USA). The purity and concentration of DNA were assessed using Thermo NanoDrop One. Specific primers, TaKaRa Premix Taq® Version 2.0 (TaKaRa Biotechnology Co., Dalian, China), and BioRad S1000 (Bio-Rad Laboratory, CA) were used for PCR amplification. The primers and PCR conditions are listed in the Supplementary document. One percent agarose gel electrophoresis was used to determine the concentration of PCR products. E.Z.N.A.® Gel Extraction Kit (Omega, USA) was used to recycle PCR products. The library was built per the standard process of NEBNext® Ultra™ II DNA Library Prep Kit for Illumina® (New England Biolabs, USA). The amplicon library was sequenced using the Illumina Nova 6,000 platform in the PE250 mode.

Paired-end raw reads were processed using fastp (an ultra-fast all-in-one FASTQ Preprocessor, v 0.14.1)^1^ and clipped with sliding window quality (− W 4- M 20). The primers were removed to obtain paired-end clean reads using cutadapt software.^2^ Non-compliant tags were filtered out by usearch fastq_ Mergepairs (V10),^3^ setting the minimum overlay length to 16 bp. To obtain the raw tags, the maximum mismatch allowed in the overlay area of the splicing sequence was 5 bp. To obtain clean tags, fastp (FASTQ Preprocessor, v0.14.1) (see Footnote 1) was used to perform sliding window quality trimming (− W 4- M 20). UPARSE was used to obtain an operational taxonomic unit (OTU) matrix (Edgar, 2013) with a cut-off of 97% (Ding et al., 2023). FastTree^4^ was used to construct the evolutionary trees. This section was performed by Guangdong Magigene Biotechnology Co., Ltd. (Guangzhou, China). The raw data on soil archaea, bacterial, fungal, nematode, and protist communities were deposited in NCBI with project PRJNA1035293 (Accession number: SAMN38085877—SAMN38085951).

### Definitions of soil rare taxa

Multivariate cut-off level analysis (MultiCoLA.1.4) was to determine the specific cut-off of rarities in relative abundance (Gobet et al., 2010). By doing so, the cut-offs of 0.1, 0.05, 0.1, 0.1, and 0.2% were used to partition the abundant and rare archaea, bacteria, fungi, nematodes, and protists in a sample (locally) and across samples (regionally) (Wang et al., 2022b), respectively.

### Species richness and assembly process of soil subcommunities

To understand the taxonomic diversity, the species richness (number of species) at the OTU level was calculated (Li et al., 2019, 2022a).

The rare and abundant microbial community assembly processes were determined using a null model (Zhou and Ning, 2017; Wan et al., 2021). The β-nearest taxon index (βNTI) was calculated to differentiate the deterministic and stochastic processes. An absolute value of βNTI >2 indicates that the deterministic process dominants microbial community succession, however, an absolute value of βNTI >2 indicates that the stochastic process dominants. βNTI <−2 indicates homogeneous selection, and, βNTI >+2 indicates variable selection. The processes of dispersal limitation, homogenizing dispersal, and drift were examined by calculating the variation between the observed Bray-Curtis distances based on the RaupCrick metric (RCbray) and the null model of RCbray. An absolute value of βNTI <2 and RCbray > +0.95 indicates dispersal limitation, and an absolute value of βNTI <2 and RCbray < −0.95 indicates homogenizing dispersal, an absolute value of βNTI<2 and an absolute value of RCbray < +0.95 indicates drift (Zhou and Ning, 2017; Cao et al., 2022).

### Ecosystem multifunctionality and functional trade-off

Aboveground multifunctionality was calculated based on aboveground functions, and underground multifunctionality was calculated based on underground functions. The entire ecosystem multifunctionality was based on both aboveground and underground functions in Section “Individual functions.”

Before calculation, the ratio of OC to TN, the ratio of OC to TP, the ratio of TN to TP, the unprotective organic carbon, the unprotective organic carbon ratio, the relative abundance of fungal plant pathogens, the relative abundance of pathotrophic fungi, and the relative abundance of twenty-two antibiotic resistance genes were reflected by using r (f) = −f + max (f), such that a high value was equal to a good state (Lefcheck et al., 2015; Ding et al., 2023). The reflection of these functions was based on the following prior knowledge: (1) high ratios of OC to TP (242.11–629.44) and the ratio of TN to TP (14.19–36.35) in the average level (32, 4) of China’s humid subtropical soil indicate an intensified P limitation (Ding and Wang, 2021); low ratio of OC to TN facilitates to high multifunctionality (Lucas-Borja and Delgado-Baquerizo, 2019); (2) high unprotective organic carbon and high unprotective organic carbon ratio were not conducive to the storage of organic carbon; (3) high relative abundance of fungal plant pathogens and pathotrophic fungi exhibited adverse effects on plant health and productivity (Fan et al., 2020); and (4) high relative abundance of the antibiotic resistance gene was a potential threat to human health (Chen et al., 2022). The “standardizeUnitScale” function in the R “multifunc” package (Byrnes et al., 2014) was used to standardize the functions. Five complementary approaches [averaging approach-based multifunctionality, entropy-based multifunctionality (at the maximum number and at each number of functions included), and threshold approach-based multifunctionality] were used to quantify the multifunctionality index for each sample (Ding and Wang, 2021; Wang et al., 2022b; Ding et al., 2023; Yang et al., 2023a). These indices cover different aspects of the multifunctionality (Zhang et al., 2023) and have been widely used in recent studies (Lucas-Borja and Delgado-Baquerizo, 2019; Fernández-Guisuraga et al., 2022; Yang J. et al., 2022; Li et al., 2022c; Wang et al., 2023a).

There are four methods for measuring functional trade-off, including descriptive analysis, correlation, and regression analysis, root mean square deviation (Yang et al., 2023c), and the geometric distance from a dot to a straight line of 1:1 (Wang et al., 2022b). The first three methods cannot quantify the trade-off for an individual sample, but the fourth can. Therefore, the geometric distance method was used to quantify the trade-off intensity for each sample (Wang et al., 2022b).

### Data analysis

The Kruskal–Wallis rank sum test was used to test the significance of the difference among shrub expansion stages, and the Wilcoxon test was used to test the differences between stages in R v3.5.3.

Pearson correlation, Random Forest (Jiao et al., 2020), Redundancy Analysis (RDA), forward selection (Dong et al., 2023), and hierarchical partitioning (Lai et al., 2022) with 999 permutations were used to determine the effects of the driving forces on the community assembly processes of soil microbiome, multifunctionality, and functional trade-off intensity. The R “vif” function was used to identify variables with collinearity. A constraint variable was excluded if the variance inflation factor of the constraint variable was ≥10 (Ding et al., 2020). Partial least squares path modeling (He et al., 2023) was used to test the potential causal pathways that could account for how shrub expansion alters the community assembly processes of soil microbiome, multifunctionality, and functional trade-off intensity using the “plspm” package in R (Sanchez, 2013). *p* < 0.05 indicates significant. The R packages “ggpubr” and “ggplot2” were used for the visualization of the results (Ding and Wang, 2021; Wang et al., 2022b).

## Results

### Shrub expansion altered ecosystem functions and functional trade-offs

**Figure 1:**
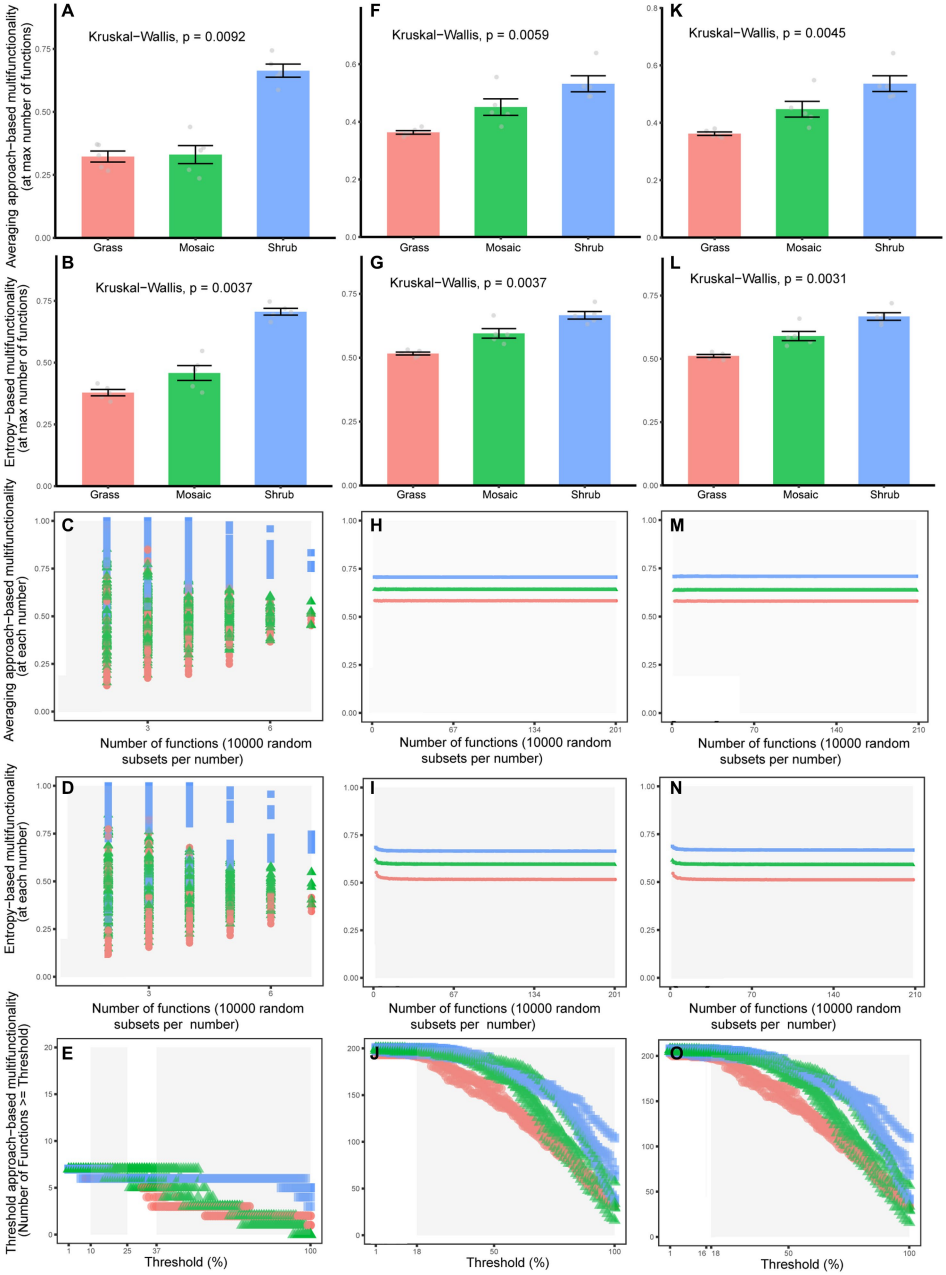
The averaging approach-based multifunctionality **(A,F,K,C,H,M)**, entropy-based **(B,G,L,D,N,I)** multifunctionality [**(A,F,K,B,G,L)** considering maximum number of functions; **(C,H,M,D,N,I)** considering the increasing number of functions], and threshold approach-based multifunctionality **(E,J,O)** of the aboveground **(A–E)**, underground **(F–J)**, and entire ecosystems **(K−O)** under different shrub expansion stages.

**Figure 2:**
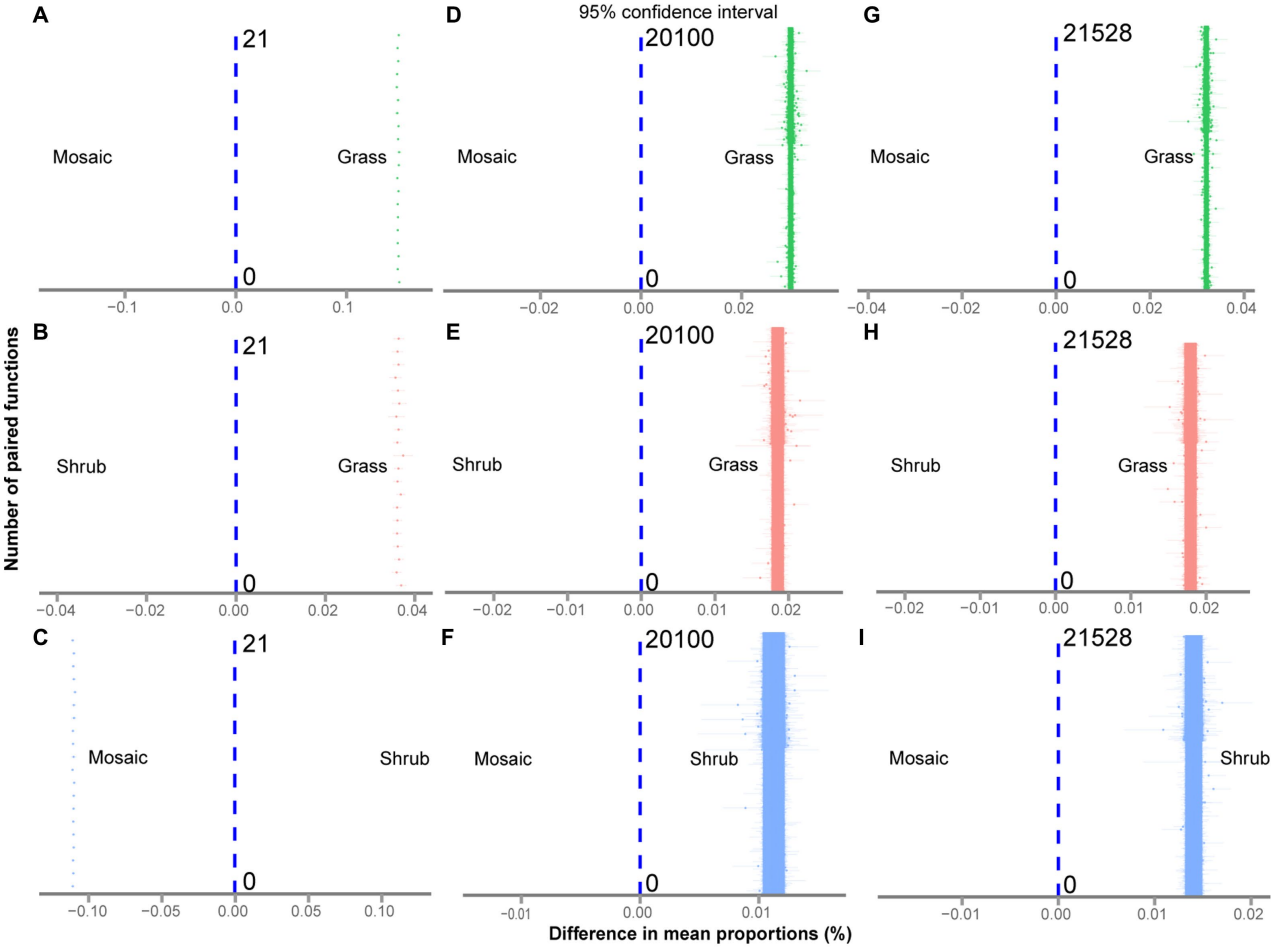
**(A−I)** Differences in the trade-off intensity of the aboveground **(A,B,C)**, underground **(D,E,F)**, and entire ecosystems **(G,H,I)** between the shrub expansion stages at the number of paired functions (*t*-test at *α* = 0.05).

The Kruskal–Wallis rank sum test showed that shrub expansion significantly enhanced five aboveground functions (*p* = 0.0018–0.0087) and significantly reduced two aboveground functions (*p* = 0.0019–0.0153, Supplementary Figure S1A; Supplementary Table S1). Moreover, shrub expansion significantly enhanced sixty-six underground functions (*p* = 0.0019–0.0356) and significantly reduced eighteen underground functions (*p* = 0.0019–0.0493, Supplementary Figure S1B; Supplementary Table S2). Twenty-three underground functions (*p* = 0.0019–0.0324) exhibited V-shaped changes with shrub expansion; however, thirty-two underground functions (*p* = 0.0024–0.0498, Supplementary Figure S1B; Supplementary Table S2) exhibited inverted V-shaped changes with shrub expansion. Five quantification methods of multifunctionality showed that shrub expansion significantly enhanced aboveground (*p* = 0–0.047, Figures 1A–E; Supplementary Tables S3–S10), underground (*p* = 0–0.043, Figures 1F–J; Supplementary Tables S11–S18) and entire ecosystem (*p* = 0–0.049, Figures 1K–O; Supplementary Tables S19–S26) multifunctionality. The functional trade-off intensity of the aboveground (*p* = 0–6e-261, Supplementary Figures S2A–C; Figures 2A–C; Supplementary Tables S27, S28), underground (*p* = 0–6e-261, Supplementary Figures S2D–F; Figures 2D–F; Supplementary Tables S29, S30), and entire ecosystems (*p* = 0–6e-261, Supplementary Figures S2G–I; Figures 2G–I; Supplementary Tables S31, S32) exhibited significant V-shaped changes with shrub expansion.

### Shrub expansion altered the assembly mechanism and species diversity pattern of the soil microbiome

**Figure 3:**
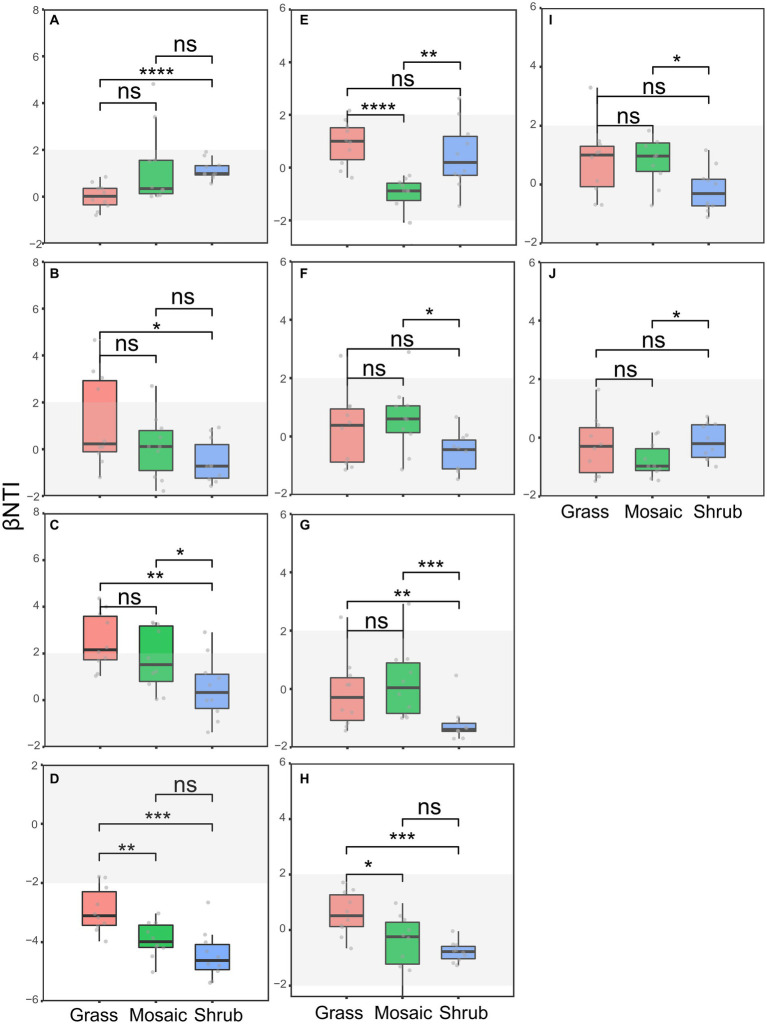
**(A−J)** Differences in the βNTI (Beta nearest taxon index) of the soil microbiomes between the shrub expansion stages. **(A)** Entire archaea subcommunity, **(B)** locally rare archaea subcommunity, **(C)** locally abundant fungi subcommunity, **(D)** locally rare fungi subcommunity, **(E)** locally abundant protist subcommunity, **(F)** entire nematode community, **(G)** locally abundant nematode subcommunity, **(H)** locally rare nematode subcommunity, **(I)** regionally abundant nematode subcommunity, **(J)** regionally rare nematode subcommunity. ns, *p* > 0.05; *, *p* < 0.05; **, *p* < 0.01; ***, *p* < 0.001; ****, *p* < 0.0001.

The phylogenetic correlogram revealed significant (*p* < 0.05) phylogenetic signals across short phylogenetic distances (Supplementary Figure S3), suggesting that the ecological traits influencing microbiome community assembly processes were phylogenetically conserved. These results allowed for the subsequent dissection of the community assembly processes. The significant differences among the shrub expansion stages (Figures 3A–J) were detected using the βNTI values (*p* = 0.0004–0.042), supporting our first hypothesis (H1). Null models based on Raup–Crick showed that shrub expansion suppressed the variable selection of the locally abundant archaea, locally rare archaea, locally abundant bacteria, locally abundant fungi, entire nematodes, locally abundant nematodes, regionally abundant nematodes, and regionally rare protists. It suppressed the homogenizing dispersal of the entire fungi, locally rare fungi, regionally rare fungi, entire protists, and locally abundant protists. It also suppressed the drift of regionally abundant fungi. Besides, shrub expansion enhanced the drift of the entire archaea, locally rare archaea, and regionally rare archaea. It enhanced the homogenizing dispersal of locally abundant bacteria and locally abundant fungi. Moreover, it enhanced the variable selection of regionally abundant archaea, regionally abundant fungi, and regionally abundant protists. It also enhanced the homogeneous selection of locally rare fungi (Supplementary Figure S4).

Shrub expansion altered the species diversity (indicated by species richness) of the soil microbiome (Supplementary Table S33). The species richness of locally abundant archaea (ANOVA, *p* = 0.033) and regionally abundant archaea (ANOVA, *p* = 0.0071) exhibited V-shaped changes with shrub expansion; however, the species richness of the entire protists (ANOVA, *p* = 0.019), regionally abundant nematodes (ANOVA, *p* = 0.011), and regionally rare nematodes (ANOVA, *p* = 0.032) exhibited opposing changes with shrub expansion. Although the species richness of regionally abundant fungi (ANOVA, *p* = 0.042) increased with shrub expansion, the species richness of entire nematodes (ANOVA, *p* = 0.0013), locally abundant nematodes (ANOVA, *p* = 0.001), regionally abundant nematodes (ANOVA, *p* = 0.0058), regionally rare nematodes (ANOVA, *p* = 0.0092), and locally abundant fungi (ANOVA, *p* = 0.011) declined with shrub expansion.

### Disentangling the effects of driving forces on the community assembly processes of the soil microbiome

Pearson correlation and Random Forest analysis showed that plant factors and elemental limitations were important factors driving the community assembly processes of soil archaea, bacteria, fungi, nematodes, and protists (Supplementary Figure S5A). RDA, forward selection, and hierarchical partitioning (999 permutations) indicated that the litter (*p* = 0.001), shrub cover (*p* = 0.002), plant species richness (*p* = 0.021), and carbon limitation (*p* = 0.006) significantly drove the assembly processes of the soil microorganisms (Supplementary Figure S5B). Litter and shrub cover had the highest individual effects; therefore, they were identified as the two strongest driving forces of the community assembly processes of the soil microbiome.

### Disentangling the effects of driving forces on the multifunctionality and functional trade-off intensity

**Figure 4:**
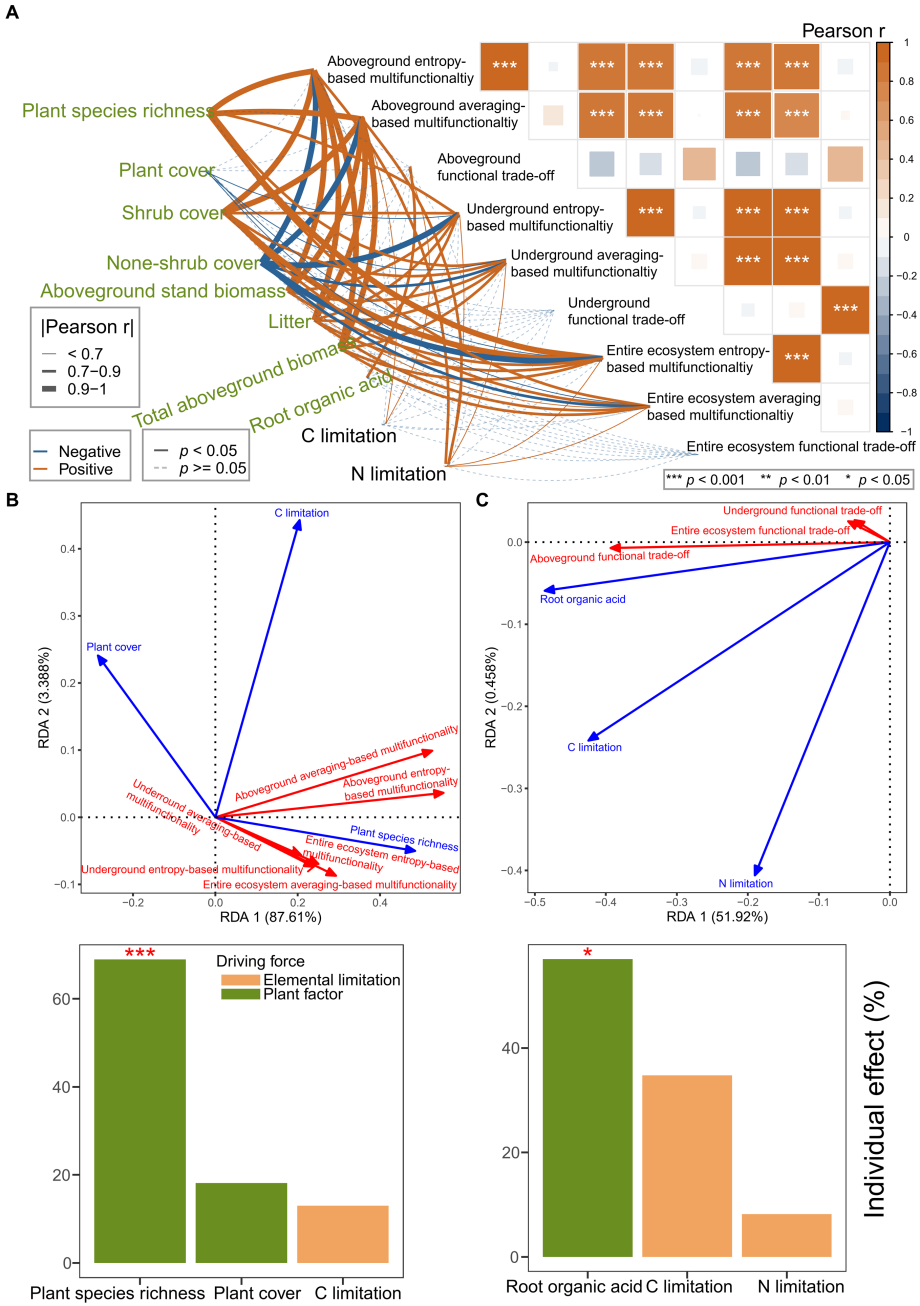
**(A–C)** Disentangling the effects of plant factors and element limitations on multifunctionality and functional trade-off intensity. **(A)** Pearson correlation analysis for the plant factors, element limitations, multifunctionality, and functional trade-off intensity. **(B,C)** RDA models showing the effects of plant factors and element limitations on the multifunctionality **(B)** and functional trade-off intensity **(C)** based on collinearity reduction, forward selection, and hierarchical partitioning with 999 permutations.

Pearson correlation analysis suggested that multifunctionality and functional trade-off intensity had closer and more frequent relationships with plant factors than with elemental limitations (Figure 4A). RDA, forward selection, and hierarchical partitioning (999 permutations) suggested that the plant factors (plant species richness, *p* = 0.001; plant cover, *p* = 0.118) had more than six times the effect than carbon limitation (*p* = 0.187) on the multifunctionality (Figure 4B). However, the plant factor (root organic acid, *p* = 0.022) had more than 30% of the effect than the elemental limitations (carbon limitation, *p* = 0.092; nitrogen limitation, *p* = 0.519) on the functional trade-off intensity (Figure 4C). Plant species richness was identified as the strongest driving force of the multifunctionality. Moreover, the root organic acids were identified as the strongest driving force of functional trade-off intensity.

**Figure 5:**
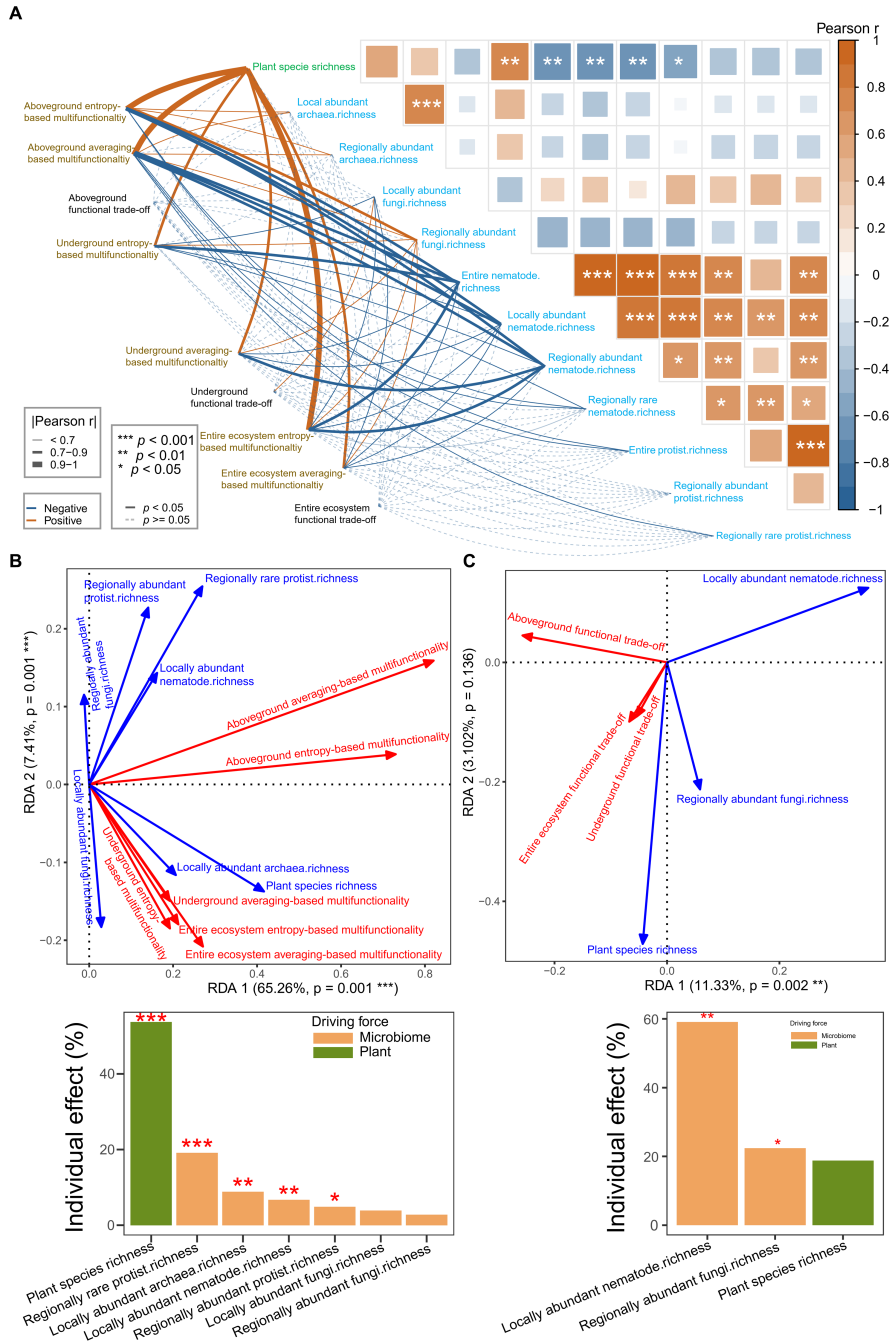
**(A–C)** Disentangling the effects of aboveground plant diversity and underground microbial diversity on multifunctionality and functional trade-off intensity. **(A)** Pearson correlation analysis for the aboveground plant diversity, underground microbial diversity, multifunctionality, and functional trade-off intensity. **(B,C)** RDA models showing the effects of aboveground plant diversity and underground microbial diversity on the multifunctionality **(B)** and functional trade-off intensity **(C)** based on collinearity reduction, forward selection, and hierarchical partitioning with 999 permutations.

Pearson correlation analysis suggested that the multifunctionality (*p* = 0–0.04892) and functional trade-off intensity (*p* = 0.0425) were significantly related to aboveground plant and underground microbial diversities (Figure 5A). Furthermore, RDA, forward selection, and hierarchical partitioning (999 permutations) showed that the plant species richness (*p* = 0.001) and the species richness of regionally rare protists (*p* = 0.001), locally abundant fungi (*p* = 0.07), locally abundant nematodes (*p* = 0.017) and locally abundant archaea (*p* = 0.004) had significant effects on the multifunctionality (Figure 5B). Moreover, the aboveground plant species richness had more than 16% effect than soil microbiome diversity did on the multifunctionality. However, only the soil microbiome diversity (locally abundant nematode richness, *p* = 0.002; regionally abundant fungi richness, *p* = 0.048) had significant effects on the functional trade-off intensity (Figure 5C). The effects of aboveground plant species diversity (plant species richness, *p* = 0.063) were just one-fifth of that of soil microbial diversity.

## Discussion

### Shrub expansion altered the community assembly processes of the soil microbiome

**Figure 6:**
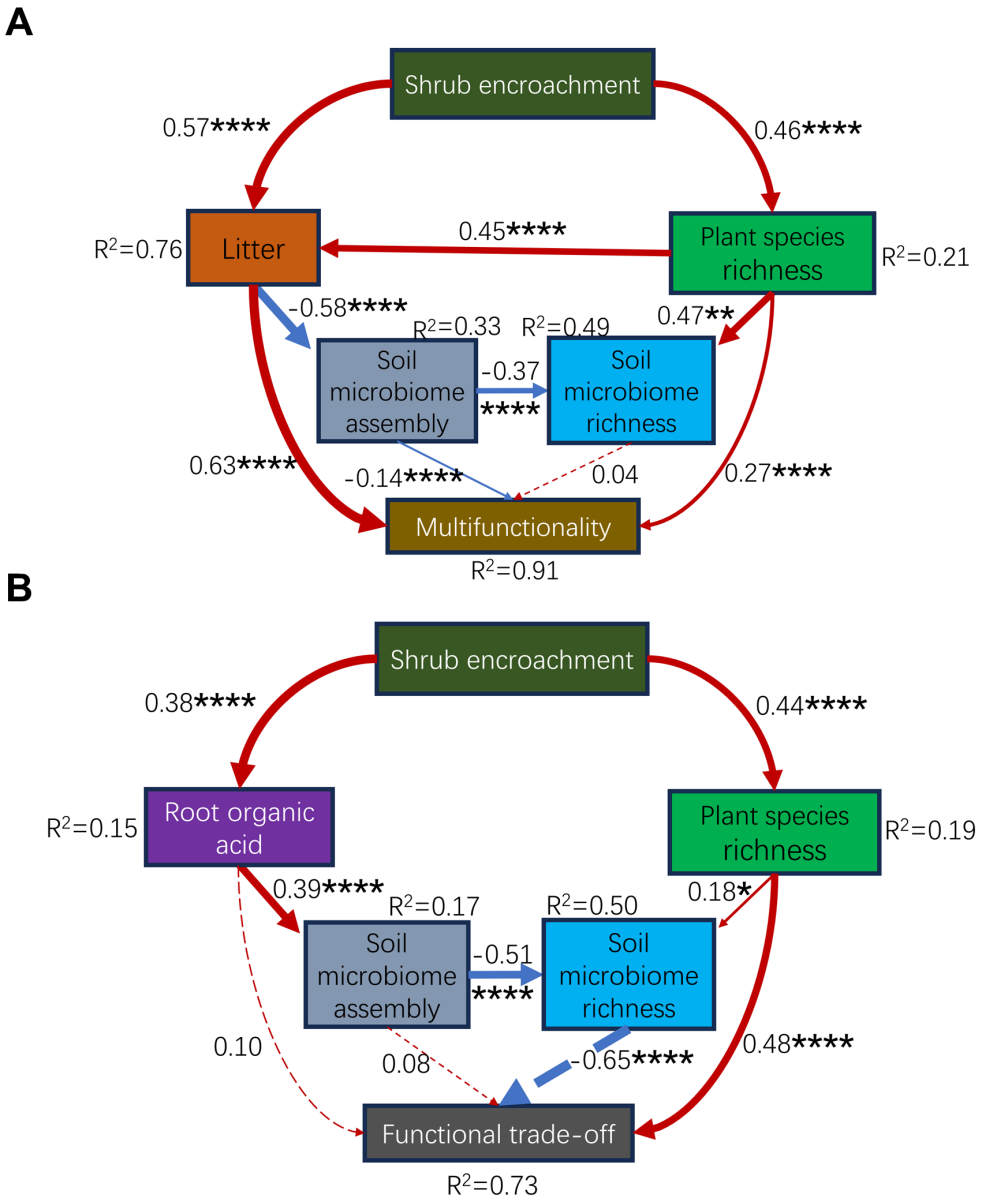
Partial least squares path modeling (PLS-PM) illustrating the cascading effects from plants to soil microorganisms, explaining how shrub expansion affects multifunctionality **(A)** and trade-offs **(B)** via plant and soil microorganisms. The goodness-of-fit for the models was 0.36 **(A)** and 0.21 **(B)**. The numbers adjacent to the arrows indicates the effect size. The red and blue lines indicate positive and negative effects, respectively. The solid and dashed lines indicate significant and nonsignificant effects, respectively. *R*^2^ denotes the proportion of the variance explained. *, *p* < 0.05; **, *p* < 0.01; ***, *p* < 0.001; ****, *p* < 0.0001.

Ecological succession and microbial community assembly are two main topics in microbial ecology (Dini-Andreote et al., 2015). In this study, the community assembly processes of the soil microbiome were changed by the succession of shrub expansion (Figure 3; Supplementary Figures S4, S6). Our previous study revealed the impact of environmental selection on soil bacteria and fungi (Ding et al., 2020). In this study, environmental selectivity, such as litter, shrub cover, plant species richness, and carbon limitation (often considered as environmental selection factors), affected the microbial community assembly (Supplementary Figure S5B). Nevertheless, the explanatory power of the environmental selection for the assembly processes was much lower than 50% (Supplementary Figure S5B). This was likely because the majority of subcommunities (21/25) were dominated (60–100%) by stochastic processes, except for the assembly of the entire bacteria, locally rare bacteria, regionally rare bacteria, and locally rare fungi, which were dominated (100%) by deterministic processes (Figure 3; Supplementary Figures S4, S6). This study provides novel findings concerning the soil microbial community assembly subjected to shrub expansion. Moreover, the best partial least squares structural equation model (Supplementary Figure S7) supported that shrub expansion mainly affected soil microbial assembly processes through litter (effect size = −0.52, *p* = 2.95e-08) rather than elemental limitations (effect size = 0.16, *p* = 6.14e-02). Previous studies have indicated that community assembly processes are dependent on environmental variables (Jiao and Lu, 2019), e.g., pH, temperature (Tripathi et al., 2018; Jiao and Lu, 2019; Shi et al., 2022), and precipitation (Yang L. et al., 2022). However, our results might go further than these findings. Shrub encroachment-induced increases in plant diversity (Zhao J. et al., 2023) increased the quantity of plant litter (Supplementary Figure S1; Figure 6A) inputs to the soil (Liu J. et al., 2023; Zhao J. et al., 2023). On the one hand, litter input caused a profound effect on soil nutrients and microorganisms (Zhao Y. et al., 2023). Litter can provide carbon and nutrients for microorganisms (Yannarell et al., 2014) in the topsoil, thereby selecting microorganisms through deterministic processes, resulting in succession within the soil community (Yannarell et al., 2014). However, only dispersal from above and near the topsoil, which was covered by litter, impacted the microbial composition (Walters et al., 2022). The litter covering the topsoil may have enhanced the stochastic processes of the nine subcommunities and weakened the eight subcommunities by changing the homogenizing dispersal (Figure 3; Supplementary Figure S4). In this sense, our findings improve the understanding of the impact of environmental factors, such as litter, on the assembly processes of neighboring soil microorganisms. Collectively, shrub expansion affected the assembly processes of soil communities by increasing the quantity of litter due to the increase in plant species richness. Changes in the assembly processes may influence the richness of soil microbial species and, in turn, modify ecosystem functionality.

### The effects of plant and soil microbial subcommunities on the multifunctionality and functional trade-offs

Recent studies have begun to reveal the relative importance of aboveground and underground biodiversity in ecosystem functioning (Cui et al., 2022; Zhang et al., 2023). In this study, the multifunctionality of the aboveground, underground, and entire ecosystems were significantly correlated with both aboveground and underground species richness (Figures 5A,B). These findings suggest that both aboveground and underground biodiversity supported ecosystem functions. This supported the results of previous studies (Valencia et al., 2018; Guo Y. et al., 2021; Wan et al., 2021; Li Z. et al., 2022; Kou et al., 2023). In line with findings from a large body of field observations and operational experiments (Valencia et al., 2018; Lucas-Borja and Delgado-Baquerizo, 2019; Wang et al., 2020; Guo Y. et al., 2021; Fan et al., 2023), plant diversity positively contributed to multifunctionality. Some studies have shown that microbial diversity significantly promotes multifunctionality (Delgado-Baquerizo et al., 2020; Guo Y. et al., 2021; Cui et al., 2022; Jiao et al., 2022a; Li et al., 2022a; Li Z. et al., 2022; Fan et al., 2023; Jia et al., 2023), whereas other studies have exhibited negative effects of microbial diversity (Valencia et al., 2018; Wang et al., 2020, 2021, 2023a; Li J. et al., 2023; Luo et al., 2023; Yang et al., 2023b). Furthermore, in the study regarding a semi-arid steppe grazing grassland (Wang et al., 2020), the absolute value of the slope of soil bacterial diversity toward multifunctionality was greater than that of plant diversity toward multifunctionality, implying that the role (negative) of soil bacterial diversity was greater than that (positive) of plant diversity. However, in studies regarding urban greenspaces (Fan et al., 2023) and restored grasslands (Guo Y. et al., 2021), the effect size (positive) of plant richness was greater than the effect size (positive) of soil biodiversity. Moreover, a recent study regarding dryland demonstrated (Zhang et al., 2023) that plant diversity had an impact but microbial diversity did not have an impact on multifunctionality in ungrazed grasslands, the opposite has occurred in grazed grasslands. In our study, the plant species richness (Figures 5A, 6A) and the richness of four subcommunities (i.e., locally abundant archaea richness, regionally abundant archaea richness, and regionally abundant fungi richness) positively contributed to multifunctionality. However, the richness of seven subcommunities (i.e., locally abundant fungi richness, entire nematodes richness, locally abundant nematodes richness, regionally abundant nematodes richness, regionally rare nematodes richness, entire protists richness, and regionally rare protists richness) negatively contributed to multifunctionality (Figure 5A). Clearly, our findings provide more information than ever before. Regarding the total effect, the species richness of the soil microbial subcommunities had almost no contribution to the multifunctionality (Figure 6A). This implies that the balance of the positive–negative effects of microbial subcommunities determines the impact of microbial diversity on multifunctionality, supporting a recent finding from the Helan Mountains (Yang et al., 2023b). This also informed that the role of soil microorganisms in multifunctionality is far more complex than previous cognitions. More interestingly, the effect of plant species richness on the multifunctionality was greater than that of species richness of the soil microbial subcommunities (Figures 5A,B, 6A). This result has not been explicitly reported, particularly in shrub-expanded grasslands. This also indicates that the increase in plant diversity induced by shrub expansion was a critical driving force for the state shift of ecosystem functions. The impacts of biodiversity on ecosystem functions have often been explained by two mechanisms (Loreau et al., 2001; Ding and Wang, 2021): (1) the complementarity effect, that is, functional complementarity, and (2) the selection effect, that is, the selection of some traits. The selection effect suggests that communities with high diversity are more likely to select some traits to support ecosystem functions. The complementary effect suggests that communities with high diversity have more facilitation to improve system functions (Ding and Wang, 2021). In this study, the increase in plant litter triggered by the plant species richness had the greatest positive effect (0.32) over the plant species richness (0.27) on the multifunctionality. Larger plant litter (Supplementary Figure S1A) selected with shrub expansion resulted in a greater potential in carbon and nutrient input (Liu J. et al., 2023). This finding suggests that the selection effect has a greater impact on the multifunctionality than the complementary effect. This finding is consistent with a previous finding from the littoral zone of the northern semi-arid region lake (Kou et al., 2023). Furthermore, based on the division of microbial subcommunities, this study presented more insights than previous findings. The plant species richness had the largest individual effect (positive) on the aboveground and underground multifunctionality compared to the species richness of the corresponding microbial subcommunity (Supplementary Figures S8A,B; Figure 5A), respectively. In studies regarding boreal forest ecosystems (Li et al., 2019), coastal salt marsh (Li et al., 2022a), and semi-arid grassland (Li Z. et al., 2022), fungal richness positively contributed to multifunctionality. More in-depth, in this shrub-expanded grassland, the species richness of the regionally abundant fungi had the largest individual effect on the entire ecosystem multifunctionality compared to the plant species richness (Supplementary Figure S8C) or the assembly of the corresponding microbial subcommunity (Supplementary Figure S8F). These findings are conducive to targeted management of aboveground, underground, or entire ecosystem multifunctionality. However, the potential mechanisms remain to be studied.

Interestingly, rather than the richness of the soil microbial subcommunities, the assembly processes of the soil microbial subcommunities driven by both the shrub encroachment-induced and the plant species richness-induced increases in the litter, affected the multifunctionality (Figure 6A). This finding is consistent with our second hypothesis (H2). Traditional biodiversity-ecosystem function research has focused on exploring the relationship between diversity and ecosystem functions (Loreau et al., 2001; van der Plas, 2019; Ding and Wang, 2021; Wang M. et al., 2022; Ding et al., 2023; Jia et al., 2023). We propose a new research paradigm in which complex community assembly processes can explain ecosystem functions better than simple species numbers. We quantitatively compared the explanatory power of the traditional classical paradigm and our new paradigm (Figure 6A). We proved that our new paradigm has a higher explanatory power than the traditional classical paradigm. Furthermore, recent studies have found that protists are the main indicators and determinants of plant performance (Guo S. et al., 2021) and health (Xiong et al., 2020). Protists may impact plants via top-down impacts on microbial community composition (Erktan et al., 2020), community assembly (Huang et al., 2021), plant-beneficial microbiome (Gao et al., 2019), soil-borne fungal pathogens (Ren et al., 2023), and bacteria-fungi interactions (Zhai et al., 2024). Therefore, protists can contribute to aboveground ecosystem functions. In this study, the assembly of the locally abundant protist community had the largest (and individual) effect on the aboveground multifunctionality, compared to the species richness of the corresponding microbial subcommunity (Supplementary Figure S8D). Besides, nematodes occupy several key trophic niches (bacterivores, fungivores, herbivores, omnivores, and predators) in belowground food webs (Li X. et al., 2022). Nematodes belong to higher trophic levels, while bacteria and fungi belong to lower trophic levels (Wang et al., 2019). Therefore, nematodes can dominate soil multifunctionality (Zhang et al., 2020; Du et al., 2022) through top–down predation on the rest of the microbiome. In this study, the assembly of the locally rare nematodes community had the largest (and individual) effect on the underground multifunctionality, compared to the species richness of the corresponding microbial subcommunity (Supplementary Figure S8E), respectively.

Previous studies have suggested that the trade-off between different functions prevents any community from providing high levels of multiple functions (Zavaleta et al., 2010; Xu et al., 2016). However, contrary to our expectations, functional trade-offs were found to be unrelated to multifunctionality (Figure 4A). We summarized the potential causes of this phenomenon. Functional trade-offs may include two situations: one is the inherent exclusion between certain functions; thus, increasing one aspect can cause another to decline (Chandregowda et al., 2018). The other is that the maximization of different functions requires different species richness or community compositions (Xu et al., 2016). A situation in which functional trade-offs reduce multifunctionality may more likely occur in the first scenario. In the second scenario, functional trade-offs may be independent of multifunctionality. In this sense, our findings are interpretable. The results suggest that improving ecosystem functionality by reducing functional trade-offs may not be feasible in this scenario. Previous studies have found that biotic factors can regulate trade-offs (Eldridge and Ding, 2020). Our recent study showed that the microbiome was most strongly related to the functional trade-off intensity (Wang et al., 2022b). In this study, functional trade-offs were found to be related to plant and soil microbial diversities. The effect of the species richness of the soil microbial subcommunities on the functional trade-offs was greater than that of the plant species richness (Figure 6B). However, this result contradicts our third hypothesis (H3). Interestingly, although root organic acids had a positive effect on the functional trade-offs, they did not have a direct positive effect. Instead, they functioned through a negative impact by the soil microbial assembly-driven inhibition in the species richness of the soil microbial subcommunities. Previous studies have suggested that root exudates affect the assembly of microbial communities (Zhalnina et al., 2018), trigger the priming effect, accelerate the soil organic matter turnover, and mobilize soil nutrients (Ma et al., 2022). On the one hand, this is beneficial for plant growth. However, this may not be conducive to soil organic matter accumulation. This may exacerbate functional trade-offs. However, more research is needed for this. To sum up, this is the only study to assess the effect of shrub expansion on grassland multifunctionality and functional trade-offs in Southern China. This study provides an update on the current understanding of the effect of plant expansion on ecosystem functions.

## Conclusion

The expansion of shrubs affects aboveground and underground diversity and multifunctionality. Five quantification methods of multifunctionality were used to demonstrate that shrub expansion significantly enhanced aboveground, underground, and entire ecosystem multifunctionality. The functional trade-off intensities of aboveground, underground, and entire ecosystems showed significant V-shaped changes with shrub expansion. Shrub expansion altered the assembly processes and species diversity patterns of soil archaea, bacteria, fungi, nematodes, and protists subcommunities. Rather than elemental limitations, litter dominated the assembly processes of soil microbial subcommunities. The effect of litter on multifunctionality was more significant than that of plant species richness. Moreover, rather than the species richness of soil microbial subcommunities, the assembly processes of the soil microbial subcommunity driven by both shrub encroachment-induced and plant species richness-induced increases in litter affected multifunctionality. The effect of species richness of the soil microbial subcommunity on the functional trade-off was more significant than that of plant species richness. Root organic acids had a positive effect on functional trade-offs through a negative impact on soil microbial assembly-driven inhibition of the species richness of soil microbial subcommunities. This study provides information regarding the first disentangling effects of shrub expansion on aboveground, underground, and entire ecosystem multifunctionality and functional trade-offs in Southern China.

## Data availability statement

The datasets presented in this study can be found in online repositories. The names of the repository/repositories and accession number(s) can be found in the article/Supplementary material.

## Author contributions

LD: Conceptualization, Formal analysis, Funding acquisition, Investigation, Methodology, Visualization, Writing – original draft, Writing – review & editing. HC: Investigation, Writing – review & editing. MW: Investigation, Methodology, Writing – review & editing. PW: Funding acquisition, Investigation, Methodology, Supervision, Writing – review & editing.

## Footnotes

^1^ https://github.com/OpenGene/fastp

^2^ https://github.com/marcelm/cutadapt/

^3^ http://www.drive5.com/usearch/

^4^ http://www.microbesonline.org/fasttree/
