# Supplementary material for: Shrub expansion raises both aboveground and underground multifunctionality on a subtropical plateau grassland: coupling multitrophic community assembly to multifunctionality and functional trade-off
Source: Front Microbiol. 2024 Jan 11;14:1339125. doi: 10.3389/fmicb.2023.1339125 (PMC10808678; doi:10.3389/fmicb.2023.1339125)
Supplement: Supplementary file 1 [file Data_Sheet_1.zip › Legends for Figure.S1-S8.docx]

**Legends for Figure.S1-S8**

**Figure.S1** Radar charts of individual functions (a, aboveground functions; b, underground functions). The full description of abbreviations is listed in Table.S1 and Table.S2.

**Figure.S2** Boxplots indicating that the functional trade-off intensity of aboveground (a, b, c), underground (d, e, f), and entire ecosystem (g, h, i) changed with the number of paired functions.

**Figure.S3** Phylogenetic signals showing the trait conservatism of soil microbiome for

ecosystem properties listed in the method section. Colored dots indicate significance (p < 0.05).

**Figure.S4** (a−y) The changes in community assembly processes of soil microbial subcommunities with shrub expansions.

**Figure.S5** (a) Contributions of driving forces (plant factors and elemental limitations) to the community assembly processes of soil microbial subcommunities based on Pearson correlation, Random Forest, and Hierarchical partitioning with 999 permutations. (b) Redundancy Analysis (RDA) showing the effect of driving forces on the community assembly processes of soil microbial subcommunities based on collinearity reduction, forward selection, and Hierarchical partitioning with 999 permutations. The size of the circle is proportional to the importance of the driving forces. *, p < 0.05; **, p < 0.01; ***, p < 0.001; ****, p < 0.0001

**Figure.S6** (a−o) The insignificant changes in βNTI (Beta nearest taxon index) of soil microbiome with shrub expansions. ns, p > 0.05.

**Figure.S7** Partial least squares path modeling (PLS-PM) illustrating the cascading effects from plants to soil microorganisms explain how shrub expansion affect the community assembly processes of soil microbial subcommunities via plant factors and elemental limitations. Numbers adjacent to arrows are indicative of the effect size. Red and blue lines indicate positive and negative effects, respectively. Solid and dashed lines indicate significant and nonsignificant effects, respectively. R^2^ denotes the proportion of variance explained. ***, p < 0.001; ****, p < 0.0001.

**Figure.S8** Contributions of driving forces (plant diversity vs. microbial diversity and microbial community assembly vs. microbial diversity) to the aboveground, underground, or entire ecosystem multifunctionality based on collinearity reduction, redundancy Analysis (RDA) forward selection, and Hierarchical partitioning with 999 permutations. *, p < 0.05; **, p < 0.01; ***, p < 0.001
