# Supplementary material for: Shrub expansion raises both aboveground and underground multifunctionality on a subtropical plateau grassland: coupling multitrophic community assembly to multifunctionality and functional trade-off
Source: Front Microbiol. 2024 Jan 11;14:1339125. doi: 10.3389/fmicb.2023.1339125 (PMC10808678; doi:10.3389/fmicb.2023.1339125)
Supplement: Supplementary file 1 [file Data_Sheet_1.zip › Supplementary document.docx]

**Supplementary documents**

**Supplementary description of methods**

**(1) Aboveground functions**

Aboveground functions were characterized as follows:

**Plant species richness** (Plantspeciesrichness) was recorded as the number of plant species in a 1 m × 1 m plot.

**Plant cover** (Plantcover) **and shrub cover** (Shrubcover) were obtained by visual estimation in the 1 m × 1 m plot.

**Aboveground stand biomass** (AB) of vegetation in the 1 m × 1 m plot was cut by levelling the ground, then the all **litter** covered the ground was collected. These vegetation samples were killed at 105 ℃, dried to constant weight at 65 ℃, and then weighed.

**none-shrub cover** (Noneshrubcover) = plant cover - shrub cover.

**total aboveground biomass** (TAB) = aboveground stand biomass + litter.

**(2) Underground functions**

Underground functions were characterized as follows:

**Organic carbon** (OC, g/kg) was measured by an elemental analyzer (Germany) (Ding et al., 2020a).

**Recalcitrant organic carbon** (ROC, g/kg) was measured using the following method:

The sample is air-dried through a 10-mesh sieve.

Weigh about 1.0g of sample into a cooking tube, add 20ml of 2.5mol/L sulfuric acid, and hydrolyze at 105°C for 30min (closed).

At 4500rpm, 20min, pour out the supernatant in a 50ml centrifuge tube, add 10ml of water, centrifuge, the supernatant is combined with a 50ml centrifuge tube, then add 10ml of up water, centrifugation and combine the supernatant, the residue is dried at 60 °C, the supernatant is fixed to 50ml, and the supernatant is activated carbon 1 and the TOC analyzer (vario TOC cube, Elementar, Germany) was used to determine the activated carbon 1.

After drying, add 2ml of 13mol/l sulfuric acid, shake for 10h, then add up water, dilute the sulfuric acid to 1mol/l, and then hydrolyze at 105 °C for 3h, shaking once every 1h. Then transfer to a high-speed centrifuge tube, centrifuge, transfer the supernatant to a 50ml centrifuge tube, then wash twice with UP water, 10ml each time, combine the supernatant in 50ml, set the volume with UP water, for activated carbon 2, and the TOC analyzer (vario TOC cube, Elementar, Germany) was used to determine the activated carbon 2.

Then wash the remaining soil into a 50ml centrifuge tube that has been weighed, at 3500rpm, centrifuge, pour off the supernatant, add up water, shake, centrifuge until the pH is neutral, take the supernatant, add silver nitrate, and not turbid, pour out the supernatant, leaving only the soil.

The centrifuge tube filled with soil was placed in a 60 °C oven and dried, which was recalcitrant organic carbon. After drying, the soil is ground to 100 mesh. The recalcitrant organic carbon in samples was determined using an elemental analyzer (vario TOC cube, Germany, elementar). In this study, the active organic carbon (AOC, g/kg) = OC – ROC.

**Heavy** **fraction organic carbon** (HOC, g/kg) and **light fraction organic carbon** (LOC, g/kg) were measured using the following method:

(a) Weigh 10.00g of air-dried soil samples through a 2mm sieve into a dry centrifuge tube, add 25mL of NaI solution (1.7 g/cm3) to the heavy liquid, shake it evenly by hand and shake it for 1h (250r/min), centrifuge it at 3500 rpm for 15min, filter the light components and recombinant components with a 0.45um filter membrane (the separation process is repeated 3 times, starting from the shaking of adding NaI solution), vacuum, and rinse the centrifuge tube wall with a small amount of NaI solution.

(b) Rinse with 0.01MCaCl2 solution to remove excess NaI, rinse the light components with 0.01M CaCl_2_ solution for 3 times until colorless, rinse with 0.01MCaCl_2_ solution for 5 times until colorless, and then rinse with distilled water until no precipitation is detected with silver nitrate solution, dry at 60°C, weigh, and determine the organic carbon content after grinding.

(c) Take out the dried part of the sample for analysis of organic carbon content (using external heating method). The allocation ratio of heavy fraction organic carbon was obtained by dividing the heavy fraction organic carbon content by the total soil organic carbon content, and the difference between soil total organic carbon and heavy fraction organic carbon was the light fraction organic carbon.

**Particulate organic carbon** (POC, g/kg) and **mineral-associated organic carbon** (MAOC, g/kg) were measured by the following method:

(a) Weigh 6 g of 10 mesh air-dried soil into a 50ml centrifuge tube, add 30ml (5g/L) of sodium hexametaphosphate, shake well and mix for 5min.

(b) Put it on a shaker for 90r/min and shake for 18h.

(c) The dispersed soil sample after oscillation passes through a 53um sieve (300 mesh) and is washed with distilled water, the soil sample on the washed sieve is POC, and the dispersed soil sample (<53um) through the sieve is mineral-bound organic carbon, that is, MAOC.

(d) All samples were dried at 60 °C, weighed, and ground finely, and the appropriate amount of organic carbon was measured.

**Oxidizable organic carbon** (OxidizableOC, g/kg) was measured using the following method:

According to the difference between the concentration of potassium permanganate (residual solution) after the "easily oxidized carbon" in the soil sample and the control concentration (theoretically 333mmol/L), the molar mass of potassium permanganate consumed by easily oxidized carbon in different soil samples was quantified, and then the content of easily oxidized organic carbon in each soil was calculated:

(a) Dry the soil sample naturally and pass it through a 0.25 mm sieve.

(b) At 25 °C, three soil samples containing 15 mg of carbon were taken and filled into a 100ml plastic bottle with 25 ml of 333 mmol/L potassium permanganate solution, and the sealed bottle was shaken at 25 r/min for 1 h. Blanks are the same as soil samples (soil samples are not included in blanks):

(c) After shaking, the sample was centrifuged at 2000 r/min for 5 min, and then the supernatant was taken and diluted with deionized water at 1:250.

(d) The above dilution is colorimetric on a spectrophotometer at 565nm, and the concentration range of the standard solution must include 1mg of carbon, and the carbon content of the easily oxidized soil sample can be calculated according to the consumption of potassium permanganate (every 1mmol potassium permanganate solution consumed is equivalent to oxidizing 9mg of carbon), and then the standard curve is drawn.

(e) Take 6 25ml volumetric flasks, add 0.2g of soil samples to each bottle, and then add them separately

0.00, 1.00, 2.00, 3.00, 4.00, 5.00 ml potassium permanganate standard solution, dilute to scale with distilled water, shake well. It is then processed with the above method of operation. Taking A565mm as the ordinate and the amount of potassium permanganate as the abscissa, the standard curve was used to investigate the regression equation.

**Dissolved organic carbon** (DOC, mg/kg) was determined using the following method:

(a) 10 g of fresh soil samples were taken, mixed according to the ratio of soil to water 1:5, oscillated at a speed of 250r/min for 1 h at 25 °C, then centrifuged at a speed of 15000 r/min for 10 min, the upper suspension passed through a thin filter membrane of 0·45 μm, and the method of measuring organic carbon was adopted in the subsequent steps.

(b) Take the solution with a 0·45μm thin filter membrane and put it into the cooking tube, add 5ml of 0.8mol/L 1/6K2Cr2O7 standard solution, then inject 5ml of concentrated sulfuric acid with a syringe, rotate and shake well, and add a small funnel to the cooking tube.

(c) Place the boiled soil sample in a wire cage and heat it in an oil bath (soybean oil) that has been preheated to 185 -190ºC. At this time, the temperature in the pot should be controlled at 170-180ºC, the boiling starts, the heating is accurate for 5min, and the cooling is taken out.

(d) After cooling, the solution is orange-yellow or yellow-green, wash the solution in the cooking tube into a 250ml triangular bottle with a washing bottle, so that the volume of the solution in the triangular bottle is about 60-80ml, add 3-4 drops of phthaloline indicator, titrate with 0.2mol/L FeSO4, the color of the solution changes as: orange-yellow→ blue-green→ brown-red, and record the amount of ferrous sulfate (V).

(e) For each batch of analytical samples, 2-3 blanks should be made; 0.1-0.5g of quartz sand was used for blank calibration instead of soil samples, and other steps were exactly the same as when measuring soil samples, and the amount of ferrous sulfate (V0) was recorded.

DOC(g/kg）=$\frac{\frac{0.8\times5.0}{V0}\times(V0-V)\times0.003\times1.1}{m1\times K2}\times1000$

where:

0.8—1/6 concentration of K2Cr2O7 standard solution (mol/L);

5.0—1/6 volume of K2Cr2O7 standard solution (ml);

V0—volume of ferrous sulfate solution for blank calibration (ml);

V—volume of ferrous sulfate solution for titrating soil samples (ml);

0.003—1/4 molar mass of carbon atom (g/m mol);

1.1—oxidation correction coefficient;

m—mass of air-dried soil sample (g);

K2—Convert air-dried soil to dried soil coefficient.

**Microbial biomass carbon** (MBC, mg/kg) was measured by chloroform fumigation extraction-elemental analyzer.

**Mineralizable carbon** (MineralizableC, g/g soil /week) was measured by short-term soil culture method, as follows:

(a) Soil pre-culture: weigh an appropriate amount of soil samples and place them at room temperature for several days to restore the soil to room temperature.

(b) Closed culture: Weigh 10g of the sample restored to room temperature and put it in a 250ml wide-mouth stopper bottle, and build a small glass bottle containing 10ml of 0.1mol/l sodium hydroxide solution, adjust the soil moisture to 60% of its maximum water holding capacity with distilled water, and incubate at a constant temperature of 5 °C for 7 days.

(c) Determination: After the end of the culture, take out the small glass bottle containing sodium hydroxide, add 2ml of barium chloride solution first, then add 2 drops of phenolphthalein indicator, and then titrate with 0.05mol/l hydrochloric acid until the red disappears, record the titration volume, calculate the release of CO2, and make a blank control (blank = water).

**Unprotective** **organic carbon ratio** (UnprotectiveOCratio) = (the average value of the proportion of POC and LOC) × 100,

**unprotective organic carbon** (UnprotectiveOC) = OC × UnprotectiveOCratio/100,

**protective organic carbon** (ProtectiveOC) = OC – UnprotectiveOC,

**the rate of conversion of** **unprotective organic carbon to protective organic carbon (K)** = protective organic carbon/ unprotective organic carbon,

**dissolved organic carbon ratio** (DOCratio) = OC/DOC × 100,

**oxidizable organic carbon ratio** (OxidizableOCratio) = OxidizableOC/OC × 100,

**microbial biomass carbon ratio** (MBCratio) = MBC/OC × 100,

**mineralizable carbon ratio** (MineralizableCratio) = MineralizableC/OC × 100,

**carbon activity** (CA) = AOC/ROC × 100.

**microbial biomass nitrogen** (MBN, mg/kg) was measured by chloroform fumigation-ICP method.

**microbial biomass phosphorus** (MBP, mg/kg) was measured by the following method:

Weigh 5~10g of soil samples (equivalent to 5g of dry soil, take part of the sample to measure the water content, and determine the weight of the soil sample) 2 parts, respectively, into a 25ml small beaker (petri dish). Put a beaker containing a sample of soil into a vacuum dryer, and place a 100ml beaker containing no ethanol chloroform (about 2/3 of the beaker), a small amount of zeolite in the beaker, and a small beaker containing 1mol/L NaOH solution (absorb the CO_2_ released during the fumigation process).

Close the lid of the vacuum dryer and vacuum with a vacuum pump to keep the chloroform boiling for 5min. Close the vacuum drying valve and incubate at 25 degrees in the dark for 24 h. The other sample is treated as a non-fumigation control in another dryer.

After the fumigation is over, open the vacuum dryer valve (the sound of air entering should be heard, otherwise the fumigation is incomplete, redo, remove the small beaker containing chloroform (reusable) and dilute NaOH solution, clean the dryer, and repeatedly vacuum (5 to 6 times for 30 minutes each, it is best to completely open the dryer lid after each vacuum) until the soil is free of chloroform taste.

Take out the fumigated and unfumigated soil samples from the dryer, transfer the soil samples to a 50ml polyethylene centrifuge tube (plastic bottle), add 40ml of 0.5mol/L NaHCO3 extract (soil-water ratio 1:8), shake fully for 30 minutes (300 r/min), and filter with slow quantitative filter paper. It is best to analyze the soil extract immediately, or freeze it at -20°C, and thaw and shake it well before use.

Blank solution preparation

Take 10 ml of NaHCO3 extract as a blank.

Specimen determination

Take an appropriate amount of the extract and then use molybdenum-antimony anti-colorimetric method to determine (the same as available phosphorus) or take an appropriate amount of the extract to dilute 10 times, add 1ml of HCL acidification and directly use ICP-OES to determine the total phosphorus content in the extract.

Soil microbial biomass phosphorus: MBP=EPT/KP

where:

EPT is the difference between fumigated and unfumigated soils;

KP is the conversion coefficient and the value is 0.4.

**Total nitrogen** (TN, g/kg) was determined by semi-micro Kjeldahl method (Bao, 2000).

**Total** **phosphorus** (TP, g/kg) was determined by NaOH digestion followed by ammonium-molybdate colorimetry (Bao, 2000; Ding et al., 2020b).

**Total potassium** (TK, g/kg) was analysed by caustic and acid dissolving method. The soil sample was melted with a strong alkali, the insoluble silicate was decomposed into soluble compounds, and the potassium in the soil mineral lattice was transformed into a soluble potassium form.

**Available nitrogen** (AN, g/kg) was determined using alkaline hydrolysis-diffusion method (Bao, 2000),

**available phosphorus** (AP, mg/kg) was the NaHCO3-ultraviolet spectrometer (Bao, 2000),

**available potassium** (AK, g/kg) was extracted with ammonium acetate (Bao, 2000).

**Inorganic carbon** (estimated as calcium carbonate, IC, g/kg) was determined using hydrochloric acid and sodium hydroxide titration. The detailed information was as follows:

Weigh 3.0 g to 10.0 g of air dried soil samples (containing 0.2g to 0.4g of calcium carbonate) passing through a 0.149 mm sieve, place them in a 100mL tall beaker, add 20.00mL of hydrochloric acid standard solution (reagent 1), stir with a glass rod to drive out the resulting CO ₂, cool, and transfer to a 100mL volumetric flask. Rinse the soil sample and beaker 5-6 times with cold water, and add water to shake to volume. Suck 50mL of clear solution into a 150mL triangular flask, add two drops of phenolphthalein indicator, and titrate with sodium hydroxide standard solution (reagent 2) to a clear red color as the endpoint. Record the number of milliliters of sodium hydroxide standard solution used.

Soil nutrient stoichiometry was expressed as molar ratios (Ding et al., 2020b), i.e., **the ratio of OC and TN** was expressed as OC__TN, **the ratio of OC and TP** was expressed as OC__TP, **the ratio of TN and TP** was expressed as TN__TP.

**Exchangeable calcium** (E_Ca, cmol(1/2 Ca2+)/kg) and **Exchangeable magnesium** (E_Mg, cmol(1/2 Ca2+)/kg) were determined by ammonium acetate exchange-EDTA complexation titration.

(a) Weigh 2.0 g of air-dried sample through 2mm sieve hole, weigh 5.0 g of light soil, put it into a 100mL centrifuge tube, add a small amount of 1mol/L ammonium acetate solution along the wall of the centrifuge tube, and stir the soil sample with a rubber head glass rod to make it a uniform mud state. Add 1mol/L ammonium acetate solution to a total volume of about 60mL, stir well, and then wash the rubber head glass rod with 1mol/L ammonium acetate solution, and put the solution into the centrifuge tube.

(b) Place the centrifuge tubes in pairs on the two plates of the coarse balance and balance the mass with ammonium acetate solution. The balanced centrifuge tube was put into the centrifuge symmetrically, centrifuged for 3-5 min, the rotation speed was 3000-4000 r/min, the centrifuged clear was collected in a 250mL volumetric flask, treated with 1mol/L ammonium acetate solution for 3-5 times, and finally 1mol/L ammonium acetate solution was used to determine the exchangeable salt base.

(c) Absorb 25.00 mL of the leaching solution of 1mol/L ammonium acetate solution to treat the soil, put them into a 200 mL beaker respectively, and evaporate them at low temperature. Add 3-5 drops of 1:3 hydrochloric acid solution to the steamed beaker to dissolve the residue, add a small amount of water to scrub the inner wall of the beaker, and add water to control the total volume of the solution at about 40mL.

(d) Determination of the amount of calcium and magnesium in one of the parts: neutralize the solution with 1:1 ammonia water to neutral (check with pH test paper), add 3.5 mL of pH10 ammonia buffer solution, add 0.1 g of K-B indicator, titrate to pure blue with 0.01 mol/L EDTA standard solution, and record the volume of consumed EDTA solution (V1). At the same time, a blank test was performed to record the volume (V0) of the EDTA solution.

**Dithionite-extractable Fe** (D_Fe_2_O_3_, g/kg), **dithionite-extractable Al** (D_Al_2_O_3_, g/kg), **organically complexed Fe oxides** (OC_Fe_2_O_3_, g/kg), **organically complexed Al oxides** (OC_Al_2_O_3_, g/kg), **poorly crystalline Fe oxyhydroxides** (PC_Fe_2_O_3_, g/kg), and **poorly crystalline Al oxyhydroxides** (C_Al_2_O_3_, g/kg) were measured using the method described in the article of Chen et al. (2021).

**pH** was measured by a pH meter (Ding et al., 2020a) with 1:2.5 suspension (w/v).

**Soil bulk density** (BD,g/cm3), **water content** (WC, %), **total porosity** (Totalporosity, %), **capillary porosity** (CP, %), **non-capillary porosity** (NCP, %) were determined according to the following method:

Place the ring knife vertically on the ground surface, use a sharp knife to gently cut the soil around the outer wall of the ring knife, and gently press the ring knife to make the ring knife slowly enter the soil. After the ring knife is completely filled with soil, use a sharp knife to gently scrape off excess soil along the upper edge of the ring knife, so that the soil surface in the ring knife is flush with the upper edge of the ring knife. cover Bottom mesh and bottom. Wet weigh the ring knife and the sample inside the ring knife, and recorded as C. Remove the upper cover and bottom cover of the weighed ring knife (be sure to keep the bottom net), and put it in a flat-bottomed plastic basin or other container, add water to the upper edge of the ring knife in the container (the water surface must not exceed the upper edge of the ring knife), and pay attention to adding water to maintain the water level, place for several hours, the soil in the ring knife fully absorbs water until saturated, take the ring knife out of the container, quickly wipe the water outside the ring knife, cover the top cover and bottom cover, and weigh the saturated weight D. Open the top cover and bottom cover of the ring knife (be sure to keep the bottom net), place the ring knife on the bracket to let the gravity water in the soil drain out, and in this process, the top cover of the ring knife should be virtually covered on the ring knife to prevent the evaporation of water in the ring knife, but the top cover can not be tightly covered. After standing for 12 hours, the top cover and bottom cover are covered and weighed as E. After weighing, the top cover and bottom cover of the ring knife are opened, and they are put into the 105°C oven to dry to constant weight, and the temperature in the oven is reduced to room temperature after drying. Open the oven, take out the ring knife, and weigh the dry weight as F after capping the top and bottom covers. The weight of the ring knife is B, and the inner volume of the ring knife is V.

V = (3.14 × diameter ^2^)/4 × height,

**Soil bulk density** (BD, g/cm3) = (F-B)/V,

**Soil water content** (WC, %) = (C-F)/(C-B) × 100,

**Soil total porosity** (Totalporosity, %) = (D-F)/V × 100,

**Soil capillary porosity** (CP, %) = (E-F)/V × 100,

**Soil non-capillary porosity** (NCP, %) = (D-E)/V × 100.

**Root biomass** (g) was measured by the following method:

The root in 5 soil cores was collected, and killed at 105 ℃ after measuring 7 **root morphology indicators** [root length (Length), root surface area (Surfacearea), root average diameter Average_diameter), root length per root volume (LenPerVol), root volume (RootVolume), root tips (Tips), and root forks (Forks)] using WinRHIZO Pro2016 (REGENT, Canada), dried to constant weight at 65 ℃, and then weighed as root biomass (Lugli et al., 2019).

**Root acid phosphatase** (umol/d/l) **and root organic acid** (%) were determined according to the methods described in the study of Wen et al. (2019) with some modified. The root sheaths of all roots in the 5 soil cores were washed several times with 100 ml of 0.2 mM CaCl_2_ aqueous solution, and then transferred to 4 centrifuge tubes (5 ml), of which 2 tubes were dropwise supplemented with two drops of Micropur with a microbial inhibitor concentration of 0.01 g/L and two drops of concentrated phosphoric acid for the determination of organic acid content, and the remaining 2 tubes were used to determine acid phosphatase activity. Samples were stored at -20 °C until determination.

8 **indicators of fungal infection of roots** includes the hypha rate of arbuscular mycorrhizal fungi (AMFhypha), arbuscules rate of arbuscular mycorrhizal fungi (AMFarbuscules), vesicle rate of arbuscular mycorrhizal fungi (AMFvesicle), AMF infection rate of root (AMFinfectionrate), hypha rate of dark septate endophytes (DSEhypha), microsclerotias rate of dark septate endophytes (DSEmicrosclerotias), infection rate of dark septate endophytes (DSEinfectionrate), and ECM infection rate of root (ECMinfectionrate). Roots were bleached in 10% (w/v) KOH solution at 90°C for 20 min, rinsed with up water, acidified with 2% (v/v) HCl for 5 min at room temperature, and then stained with 0.05% (w/v) nonvital Trypan blue at 90°C for 30 min (Wen et al., 2019). Roots were soaked in a lactic acid-glycerol-water (v/v/v, 1:1:1) solution overnight (Wen et al., 2019). 20 - 40 root fragments (ca. 1 cm) were randomly transferred to 2-4 slides for observation with a light microscope. For a replicate, a total of 801 scopes were recorded for the presence of hyphae, vesicles and arbuscules of arbuscular mycorrhizal fungi, and the presence of hypha and microsclerotias of dark septate endophytes. The rate of a fungal structure was calculated as the presence of the fungal structure divided by 801. The infection rate calculated as the any presence of the above fungal structures divided by 801. The number of root tips infected by ECM fungi were characterized by the presence of swollen mantle (Han et al., 2021). A total of 101 scopes were observed. ECM infection rate was calculated as ECM root tip scopes divided by 101.

**Relative abundance of arbuscular mycorrhizal fungi, relative abundance of ectomycorrhizal fungi , relative abundance of symbiotroph fungi, relative abundance of fungal plant pathogen, relative abundance of pathotroph fungi** were inferred by parsing the soil phylotypes using FUNguild with only highly probable and probable guilds (Jiao et al., 2022).

22 **antibiotic resistance genes** includes soil bacterial ABC transporters (ABC_transporters), soil bacterial Aminoglycoside (Aminoglycoside), soil bacterial Bacitracin (Bacitracin), soil bacterial β-lactam (Beta_Lactam), soil bacterial Cationic antimicrobial peptide (Cationic_antimicrobial_peptide), soil bacterial Colistin (Colistin), soil bacterial Fosfomycin (Fosfomycin), soil bacterial fosmidomycin (Fosmidomycin), soil bacterial Kasugamycin (Kasugamycin), soil bacterial lincomycin (Lincomycin), soil bacterial Macrolide (Macrolide), soil bacterial Multidrug type (Multidrug), soil bacterial multidrug subtype (multidrug), soil bacterialmupirocin (Mupirocin), soil bacterial Peptidases_and_inhibitors (Peptidases_and_inhibitors), Phenicol (Phenicol), soil bacterial quinolone (Quinolone), soil bacterial sulfonamide (Sulfonamide), soil bacterial Tetracycline (Tetracycline), soil bacterial trimethoprim (trimethoprim), soil bacterial Vancomycin (Vancomycin), soil total bacterial antibiotics resistance gene (Total_ARGs). The relative abundance of these antibiotic resistance genes was obtained by using R package “TaxFun2”.

40 **plant residue indicators** includes Vanillin (Vanillin), Acetovanillone (Acetovanillone), Acetosyringone (Acetosyringone), vanillic acid, (vanillic_acid), syringaldehyde (syringaldehyde), Syringic acid (syringic_acid), p coumaric acid (p_coumaric_acid), Ferulic (ferulic), Lignin phenol (plant residue) content in soil (L), Hydroxycetic acid (Hydroxycetic_acid), Hexadecane diacid (Hexadecane_diacid), 9-10-p-hydroxy octadecane acid (X9_10_p_hydroxy_octadecane_acid), Hydroxy Octadecane-9-enolic acid (Hydroxy_Octadecane_9_enolic_acid), 18-hydroxyoctadecanoic acid (X18_hydroxyoctadecanoic_acid), Eicosane diacid (eicosane_diacid), Docosandioic acid (docosandioic_acid), Hydroxy Tetracosane acid (Hydroxy_Tetracosane_acid), Hydroxy hexadecanoic acid (Hydroxy_hexadecanoic_acid), Hydroxyoctacosanoic acid (Hydroxyoctacosanoic.acid), Hydroxytriacontanoic acid (Hydroxytriacontanoic_acid), Hydroxy Icosane acid (Hydroxy_Icosane_acid), Hydroxydocosanoic acid (Hydroxydocosanoic_acid), Keratin and xylon (plant residue) content in soil (B), N-pentadecane (N_pentadecane), Docosanol (docosanol), Docosanoic_acid (docosanoic_acid), Tetracosanol (tetracosanol), octacosane (octacosane), Lignoceric acid (lignoceric_acid), Nonacosane (Nonacosane), Pentacosanoic acid (pentacosanoic_acid), 1-Hexacosanol (X1_Hexacosanol), Hexacosanoic acid (Hexacosanoic_Acid), Triacontane (triacontane), Hentriacontane (Hentriacontane), Octacosanol (Octacosanol), Octadecanoic acid (Octadecanoic_acid), Triacontanol (Triacontanol), Triacontanoic acid (triacontanoic_acid), and free lipid (plant residue) content in soil (F). The determination of lignin phenol was as follows: Lignin was oxidized by alkaline copper oxide to release lignophenol monomers. Eight lignophenol monomers (Vanillin, Acetovanillone, Acetosyringone, vanillic_acid, syringaldehyde, syringic_acid, p_coumaric_acid, and ferulic) were then derivatized by BSTFA, and the derived derivatives could be determined by gas chromatography–mass spectrometry (Otto et al., 2005).

1. Oxidation: weigh about 0.5-1.0 g of soil samples in the tetrafluoroethylene reaction kettle, and then add 1 g of copper oxide, 0.1 g ammonium ferrous sulfate, and 15 mL of 2 mol/L NaOH solution. Replace the air in the kettle with nitrogen for 15 min and seal it. Place in an oven at 170 °C for 2.5 h for hydrolysis.

2. Purification:

a) Acidification: After the hydrolysate is cooled to room temperature, 400μL of ethyl vanillin solution is added (accurately weigh 10 mg of ethyl vanillin in a 100 mL brown volumetric flask, and use 2mol/LNaOH to set the volume to the scale). The vortex was shaken for 30 seconds and mixed. All hydrolysates were placed in a 50 mL centrifuge tube and centrifuged at 4000 rpm for 3 min. Take the supernatant into another 50 mL centrifuge tube, wash the pellet twice with 5 mL of pure water and sonicate for 10 min, and combine the supernatant. Adjust pH <1 with 6mol/L hydrochloric acid and place in the dark for 1h.

b) Extraction: Extract with 10mL ethyl acetate, repeat 3 times, combine the extraction solution in a 30mL glass centrifuge tube, and gently nitrogen blow at 38°C until dry. Immediately dissolve the residue with 500 μL of pyridine. Transfer to a 2 mL vial and gently nitrogen-blow at 38°C until dry.

3. Standard sample preparation:

Prepare 3 standard samples at the same time. In addition, 100 μL of mixed standards (all 8 standards are 1 mg/mL of 2mol/LNaOH solubilization solution), 400 μL of ethyl vanillin solution (10 mg of ethyl vanillic acid was accurately weighed in a 100 mL brown volumetric flask, and 2mol/L NaOH was used to set the volume to the scale), gently shaken, and dried with the sample derivatization bottle.

4. Derivatization: Add 100 μL of pyridine and 400 μL of BSTFA to the derivatization flasks of the blow-dried samples and standard samples and close them tightly. Vortex for 30 seconds to mix, react at 70 °C for 3 h, and then cool down before using GC-MS.

The keratin and xylon (plant residue) was determined as follows:

Hydrolysis: weigh about 1.0~2.0g of soil samples in a tetrafluoroethylene reaction kettle, 1mol/L methanol sodium hydroxide 3mL, boiling water bath for 3h.

Cleanse:

a. Acidification: After the hydrolysate is cooled to room temperature, rinse the hydrolysis tube with 10ml of methanol:dichloromethane (1:1) mixture, and sonicate for 15min. Take the supernatant and acidify it with HCl to pH <1 and then add 15 ml of deionized water.

b. Extraction: Collect the organic phase in a 5 mL derivatization flask and gently nitrogen-blow at 38 °C until dry.

Derivatization: Add 100 uL of pyridine and 400 uL of LBSTFA to the blow-dried derivation bottle and close the cap. Vortex for 30s to mix, react at 70 °C for 3h, and then cool down before using GC-MS.

The free lipid (plant residue) was measured using the following method:

Extraction: Weigh about 0.5~1.0g of soil sample into a 10mL centrifuge tube, add 5mL of acetone:dichloromethane (1:1) mixture ultrasonic extraction for 20min, and centrifuge to collect the supernatant. Repeat twice to pool the supernatant and dry it with nitrogen.

Derivatization: Add 100 μL of pyridine and 400 μL of LBSTFA to the blow-dried samples and standard derivatization bottles and close the cap tightly. Vortex for 30s to mix well, react at 70 °C for 3h, and then cool down and measure using the GC-MS (GC 2030, MS QP2020NX, Shimadzu, Japan).

20 **microbial residue indicators** includes soil glucosamine content (glucosamine), soil aminomannose content (Aminomannose), soil galactosamine content (Galactosamine), soil muramic acid content (Muramicacid), glucosamine necromass carbon content (GluN_C), necromass carbon content in galactosamine (GalN_C), necromass carbon in muramic acid (MurA_C), fungal glucosamine necromass content (F_GluN), fungal glucosamine necromass carbon content (FungalNC), bacterial necromass carbon content (BacterialNC), the content of fungal necromass carbon and bacterial necromass carbon (FungalNC_BacterialNC), fungal necromass nitrogen content (FungalNN), bacterial necromass nitrogen content (BacterialNN), the content of fungal necromass nitrogen and bacterial necromass nitrogen (FungalNN_BacterialNN), the ratio of fungal necromass carbon to bacterial necromass carbon (FungalNC__BacterialNC), the ratio of fungal necromass nitrogen to bacterial necromass nitrogen (FungalNN__BacterialNN), the ratio of fungal necromass carbon to soil organic carbon (FungalNCratio), the ratio of bacterial necromass carbon to soil organic carbon (BacterialNCratio), the ratio of fungal necromass nitrogen to soil total nitrogen (FungalNNratio), and the ratio of bacterial necromass nitrogen to soil total nitrogen (BacterialNNratio). These four amino sugars in soil [muramic acid (MurN), glucosamine (GlcN), aminomannose (ManN), and laminogalactose (GalN)] were detected by using gas chromatography.

Hydrolysis:

1. Weigh about 0.5~1.0g (containing 0.3mgN) of air-dried soil through a 0.15mm sieve into a hydrolysis tube, add 10 mL of 6 mol/L hydrochloric acid, and place it in an oven at 105 °C for 8h hydrolysis.

cleanse

1. After the hydrolysate is cooled to room temperature, add 100 μg inositol (1 mg/mL aqueous solution, 100 μL). The vortex was shaken for 30 seconds and mixed. The hydrolysate was filtered through No. 2 slow qualitative filter paper into a 125mL vacuum filter flask.

2. Plug the mouth of the vacuum filter bottle with filtrate, take a water bath at 45°C, and vacuum drain.

3. Dissolve the residue twice with 20mL of pure water into a 50mL centrifuge tube. Add 0.4MKOH3~5mL, and then adjust the pH to 6.6~6.8 with 0.01MHCL (the liquid generates a large number of brick-red precipitates).

4. Centrifuge at 4000rpm for 10min, transfer the supernatant to a 50mL centrifuge tube, freeze-dry (about 1.5d, carefully eject) and then add 5mL of anhydrous methanol. After vortexing to dissolve the organic matter on the tube wall (the better the third step is, the less the solution will precipitate), centrifuge at 4000rpm for 10min, and remove the salt.

5. Transfer the supernatant (try not to pour into the pellet, if necessary, it can be filtered with an organic filter head) to a derivatization tube (10mL screw cap glass centrifuge tube) and dry it with nitrogen at 45°C.

6. Add 1mL of pure water and 100μg of N-methylglucosamine (1mg/mL aqueous solution, 100μL), shake well and freeze-dry.

7. Prepare 3 standard samples at the same time. In addition, 100 μL of the mixed standard (1 mg/mL glucosamine, glucogalactose, aminomannose, 0.5 mg/mL muramic acid), 100 μg inositol (1 mg/mL aqueous solution, 100 μL), 100 μg N-methylglucosamine (1 mg/mL aqueous solution, 100 μL), and 1 mL of water were added to the derivatization tube. After shaking, freeze-dry with the samples.

Derive from:

1. Add 300 μL of derivatization reagent (weigh 320mg of hydroxylamine hydrochloride and 400mg of 4-dimethylaminopyridine, dissolved and diluted to 10ml with pyridine:methanol = 4:1 (v:v) solution) to the derivatization tube of the sample and standard sample, and then close tightly. Vortex for 30 s to mix well.

2. Take a water bath at 80°C for 35min, shake once every 5min. After cooling to room temperature, 1mL of acetic anhydride was added, vortex for 30s and mixed, and then a water bath at 80 °C for 25min, shaking every 5min. After the derivatization tube was cooled to room temperature, 1.5mL of dichloromethane was added, and the mixture was mixed by vortexing for 30s.

Derivative purification:

1. Add 1mL of 1MHCL to the derivatization tube, cap and seal and vortex for 30s. After standing for stratification, the upper inorganic phase is sucked out.

2. Add 1mL of pure water and repeat the above steps 3 times. The last time try to aspirate out the inorganic phase.

3. The remaining organic phase is dried with nitrogen at 45°C. It is then dissolved to 300 μL of diluent (ethyl acetate:n-hexane = 1:1 (V:V)) and then transferred to an injection vial with an inner tube to be tested.

48 **soil enzyme activities** related to soil carbon, nitrogen, and phosphorus cycling includes soil β-Glucosidase (BGC), soil carbonic anhydrase (CAH), soil cellobiohydrolase (CBH), soil cellulase (Cellulase), soil invertase (Invertase), soil chitinase (chitinase), soil aryl sulfatase (ASF), soil aryl amidase (AAD), soil Aromatase (Aromatase), soil amylase (AMS), soil α- Galactosidase (a_GAL), soil α-Manase (a_Manase), soil β- Galactosidase (b_GAL), soil β-xylosidase (b_xylosidase), soil ammonia monooxygenase (AMO), soil hemicellulase (Hemicellulase), soil N-acetyl-β-D-glucosidase (NAG), soil leucine aminopeptidase (LAP), Soil Glutamine Synthase (GS), soil nitrogenase (nitrogenase), Nitrate reductase containing copper ions in soil (CuNaR), soil containing cytochrome cd1 nitrite reductase (Nirs), soil hydroxyammonia oxidoreductase (HAO), soil soluble cytoplasmic nitrate reductase (SCNR), soil membrane relies on nitrate reductase (NarG), soil nitrate reductase (NAR), soil nitrite oxidoreductase (NXR), soil nitrite oxidase (NIO), soil nitrous oxide reductase (N2OR), soil nitric oxide reductase (NOR), soil nitric oxide synthase (NOS), soil acid phosphatase (ACP), soil poly⁃P⁃AMP⁃phosphotransferase (PAP), soil extracellular polyphosphatase (EPP), soil polyphosphate kinase (PPK), soil polyphosphate endonuclease (PPN), soil polyphosphate esterase (PPX), soil alkaline phosphatase (ALP), soil pyrophosphatase (PPA), soil polyphosphate mannose kinase (PPMK), soil polyphosphate fructose kinase (PPFK), soil polyphosphate glucokinase (PPGK), soil phosphate monoesterase (PMS), soil phosphodiesterase (PDE), soil phosphate acetaldehyde hydrolase (PAH), soil metaphosphatase (MP), soil adenosine triphosphate enzyme (ATPase), and soil phytase (phytase). These enzyme activities were measured according to the kit instructions (Shanghai Enzyme-linked Biotechnology Co., Ltd., China) (Ding et al., 2020a).

**Microbial carbon limitation** (C_limitation) **and microbial nitrogen limitation** (N_limitation) were quantified using vector length and angle analysis based on the four enzyme activities (BGC, NAG, LAP, and ACP) (Ding et al., 2020a). The length and angle of vectors defined by ratios of enzyme activities [BG/(NAG+LAP) vs. BG/AP] represent relative microbial investments in C (length), and N and P (angle) acquiring enzymes (Moorhead et al., 2013). This method is widely used to study microbial element limitation, especially in Guizhou ecoregion (Ding et al., 2023; Ding and Wang, 2021; Guan et al., 2022). The vector length and angel analysis were performed using a R function (https://github.com/dlltargeting/evcmdl) (Ding and Wang, 2021) with a default setting “trans=="1"”.

**The primers and PCR conditions**

The archaea V4-4 was PCR-amplified using the universal primers Uni519F (5′- CAGYMGCCRCGGKAAHACC -3′) and Arch806R (5- GGACTACNSGGGTMTCTAAT -3′) (Zhang et al., 2014), the bacterial V3V4-1 was PCR-amplified using the universal primers 338F (5′- ACTCCTACGGGAGGCAGCA -3′) and 806R (5- GGACTACHVGGGTWTCTAAT -3′) (Cui et al., 2017), the fungal ITS2-1 was PCR-amplified using the universal primers ITS3 (5′- GCATCGATGAAGAACGCAGC -3′) and ITS4 (5- TCCTCCGCTTATTGATATGC -3′) (Zuo et al., 2018), the nematode 18S rRNA was PCR-amplified using the universal primers NF‐1 (5′- GGTGGTGCATGGCCGTTCTTAGTT -3′) and 18Sr2b (5- TACAAAGGGCAGGGACGTAAT -3′) (Orr et al., 2020), the protist 18S V4-1 was PCR-amplified using the universal primers T1-F (5′- CCAGCASCYGCGGTAATTCC -3′) and 981R(5- ACTTTCGTTCTTGATYRA -3′) (Hirakata et al., 2019).

The PCRs were conducted using the following programme: 5 min of denaturation at 94 °C, 30 cycles of 30 s at 94 °C, 30 s for annealing at 52 °C, 30 s for elongation at 72 °C, and a final extension at 72 °C for 10 min. PCRs were performed in triplicate using the PCR instrument (BioRad S1000, Bio-Rad Laboratory，CA) with a 50-μl mixture containing 25 μl of 2 × Premix Taq, 1 μl of Primer-F (10 μM), 1 μl Primer-R (10 μM), Nuclease-free water and 50 ng of DNA..

**Location of study area**

library(maps)

library(mapdata)

library(ggplot2)

map("china", col = "red4", xlim = c(70,140), ylim = c(18, 54), panel.first = grid())

points(x=107.1,y=26.2,pch=19, col="red")

References:

Bao SD. Soil and Agricultural Chemistry Analysis. 3rd ed. Beijing: China Agriculture Press, 2000.

Chen L, Fang K, Wei B, Qin S, Feng X, Hu T, Ji C, Yang Y, Cleland E. 2021. Soil carbon persistence governed by plant input and mineral protection at regional and global scales. Ecology Letters 24, 1018-1028.

Cui B, Liu X, Yang Q, Li J, Zhou X, Peng Y. 2017. Achieving partial denitrification through control of biofilm structure during biofilm growth in denitrifying biofilter. Bioresource Technology 238, 223-231.

Ding L, Shang Y, Zhang W, Zhang Y, Li S, Wei X, Zhang Y, Song X, Chen X, Liu J, Yang F, Yang X, Zou C, Wang P. 2020a. Disentangling the effects of driving forces on soil bacterial and fungal communities under shrub encroachment on the Guizhou Plateau of China. Sci. Total. Environ. 709, 136207.

Ding L, Tian L, Li J, Zhang Y, Wang M, Wang P. 2023. Grazing lowers soil multifunctionality but boosts soil microbial network complexity and stability in a subtropical grassland of China. Front Microbiol 13, 1027097.

Ding L, Wang P. 2021. Afforestation suppresses soil nitrogen availability and soil multifunctionality on a subtropical grassland. Sci Total Environ 761, 143663.

Ding L, Wang P, Zhang W, Zhang Y, Li S, Wei X, Chen X, Zhang Y, Yang F. 2020b. Soil stoichiometry modulates effects of shrub encroachment on soil carbon concentration and stock in a subalpine grassland. iForest - Biogeosci. Forest. 13, 65-72.

Guan HL, Fan JW, Lu X. 2022. Soil specific enzyme stoichiometry reflects nitrogen limitation of microorganisms under different types of vegetation restoration in the karst areas. Applied Soil Ecology 169.

Han M, Chen Y, Li R, Yu M, Fu L, Li S, Su J, Zhu B. 2021. Root phosphatase activity aligns with the collaboration gradient of the root economics space. New Phytologist 234, 837-849.

Hirakata Y, Hatamoto M, Oshiki M, Watari T, Kuroda K, Araki N, Yamaguchi T. 2019. Temporal variation of eukaryotic community structures in UASB reactor treating domestic sewage as revealed by 18S rRNA gene sequencing. Scientific Reports 9.

Jiao S, Lu Y, Wei G. 2022. Soil multitrophic network complexity enhances the link between biodiversity and multifunctionality in agricultural systems. Glob Chang Biol 28, 140-153.

Lugli LF, Andersen KM, Aragão LEOC, Cordeiro AL, Cunha HFV, Fuchslueger L, Meir P, Mercado LM, Oblitas E, Quesada CA, Rosa JS, Schaap KJ, Valverde-Barrantes O, Hartley IP. 2019. Multiple phosphorus acquisition strategies adopted by fine roots in low-fertility soils in Central Amazonia. Plant and Soil 450, 49-63.

Moorhead DL, Rinkes ZL, Sinsabaugh RL, Weintraub MN. 2013. Dynamic relationships between microbial biomass, respiration, inorganic nutrients and enzyme activities: informing enzyme-based decomposition models. Frontiers in Microbiology 4.

Orr JN, Neilson R, Freitag TE, Roberts DM, Davies KG, Blok VC, Cock PJA. 2020. Parallel Microbial Ecology of Pasteuria and Nematode Species in Scottish Soils. Frontiers in Plant Science 10.

Otto A, Shunthirasingham C, Simpson MJ. 2005. A comparison of plant and microbial biomarkers in grassland soils from the Prairie Ecozone of Canada. Organic Geochemistry 36, 425-448.

Wen Z, Li H, Shen Q, Tang X, Xiong C, Li H, Pang J, Ryan MH, Lambers H, Shen J. 2019. Tradeoffs among root morphology, exudation and mycorrhizal symbioses for phosphorus‐acquisition strategies of 16 crop species. New Phytologist 223, 882-895.

Zhang J, Yang Y, Zhao L, Li Y, Xie S, Liu Y. 2014. Distribution of sediment bacterial and archaeal communities in plateau freshwater lakes. Applied Microbiology and Biotechnology 99, 3291-3302.

Zuo T, Wong SH, Cheung CP, Lam K, Lui R, Cheung K, Zhang F, Tang W, Ching JYL, Wu JCY, Chan PKS, Sung JJY, Yu J, Chan FKL, Ng SC. 2018. Gut fungal dysbiosis correlates with reduced efficacy of fecal microbiota transplantation in Clostridium difficile infection. Nature Communications 9.

**Description concerning** **the** **assembly mechanism** **of** **soil** **microbiome**

The significant differences among shrub expansion stages (Figure.4a-j) were detected in the βNTI of entire archaea (Kruskal-Wallis rank sum test, p = 0.003), local rare archaea (anova, p = 0.042), local abundant fungi (Kruskal-Wallis rank sum test, p = 0.0079), local rare fungi (Kruskal-Wallis rank sum test, p = 0.0015), entire nematode (Kruskal-Wallis rank sum test, p = 0.043), local abundant nematode (Kruskal-Wallis rank sum test, p = 0.0027), local rare nematode (Kruskal-Wallis rank sum test, p = 0.0026), regional abundant nematode (Kruskal-Wallis rank sum test, p = 0.032), regional rare nematode (Wilcoxon test, p = 0.029), and local abundant protist (Kruskal-Wallis rank sum test, p = 0.0004), the βNTI of other subcommunities showed no statistical differences among stages (Figure.S3).

**Analysis progresses involved in RDA, forward selection, and Hierarchical partitioning**

1. Disentangling the effects of driving forces on soil microbiome community assembly processes

Hierarchical partitioning permutation test with 999 times suggested that plant cover (p = 0.032), shrub cover (p = 0.003), none-shrub cover (p = 0.003), aboveground stand biomass (p = 0.003), litter (p = 0.002), total aboveground biomass (p = 0.001), root organic acid (p = 0.041), carbon limitation (p = 0.011), and nitrogen limitation (p = 0.041) significantly affect the assembly of soil microorganisms. We used RDA to analyze the effects of plant factors and element limitations on the assembly process of soil microorganisms. The none-shrub cover, aboveground stand biomass, and total aboveground biomass had high variance inflation factors (value > 10) and were therefore removed from the RDA model. Furthermore, the litter (p = 0.001), shrub cover (p = 0.001), plant species richness (p = 0.009), and carbon limitation (p = 0.002) were included into the RDA model by forward selection. Individual effects of the included factors were quantified by Hierarchical partitioning with 999 permutations. Results indicated that the litter (p = 0.001), shrub cover (p = 0.002), plant species richness (p = 0.021), and carbon limitation (p = 0.006) significantly drove the assembly process of soil microorganisms (Figure.6b). Of which, the litter and shrub cover had the highest individual effects, therefore, were identified as the two strongest driving forces of soil microbiome community assembly processes.

2. Disentangling the effects of driving forces on multifunctionality and functional trade-off intensity

We used RDA to analyze the effects of plant factors and element limitations on the multifunctionality and functional trade-off intensity. The litter, total aboveground biomass, aboveground stand biomass, shrub cover and none-shrub cover had high variance inflation factors (value > 10) and were thus removed from the RDA model of multifunctionality and the RDA model of functional trade-off intensity. Furthermore, the plant species richness (p = 0.001), carbon limitation (p = 0.007), and plant cover were included into the reduced RDA model of multifunctionality, however, only the root organic carbon (p = 0.004) was included into the reduced RDA model of functional trade-off intensity, by forward selection. Even so, element limitations (carbon limitation and nitrogen limitation) were artificially incorporated into the reduced RDA model of functional trade-off intensity to quantitatively compare the effects of plant factors and element limitations. Individual effects of the included factors were quantified by Hierarchical partitioning with 999 permutations. Results indicated that the plant factors (plant species richness, p = 0.001 and plant cover, p = 0.118) had more than six times effect than carbon limitation did (p = 0.187) on the multifunctionality (Figure.7b), however, the plant factor (root organic acid, p =0.022) had more than 30% effect than elemental limitations (carbon limitation, p = 0.092 and nitrogen limitation, p = 0.519) did on functional trade-off intensity (Figure.7c). Of which, the plant species richness had the highest individual effect, therefore, was identified as the strongest driving force of multifunctionality. The root organic acid had the highest individual effect, therefore, was identified as the strongest driving force of functional trade-off intensity.

Furthermore, we compared the effects of aboveground plant diversity and underground microbial diversity on multifunctionality and functional trade-off intensity through RDA. Entire protist richness and entire nematode richness had high variance inflation factors (value > 10), were excluded from the RDA model of multifunctionality and Entire protist richness, entire nematode richness, regionally abundant nematode richness, and regionally rare protist richness had high variance inflation factors (value > 10), were excluded from the RDA model of functional trade-off intensity. Finally, the plant species richness (p = 0.001), the species richness of regionally rare protist (p = 0.001), regionally abundant protist (p = 0.001), locally abundant fungi (p = 0.001), locally abundant nematode (p = 0.003), regionally abundant fungi (p = 0.005), and locally abundant archaea (p = 0.038) were included into the reduced RDA model of multifunctionality, the locally abundant nematode richness and regionally abundant fungi richness were included into the reduced RDA model of functional trade-off intensity, through forward selection. However, the plant species richness was artificially incorporated into the reduced RDA model of functional trade-off intensity to quantitatively disentangle the effects of aboveground plant species diversity and underground soil microbiome diversity. Hierarchical partitioning with 999 permutations showed that the plant species richness (p = 0.001), the species richness of regionally rare protist (p = 0.001), locally abundant fungi (p = 0.07), locally abundant nematode (p = 0.017) and locally abundant archaea (p = 0.004) had significantly effects on multifunctionality (Figure.8b) and the aboveground plant species richness had more than 16% effect than underground soil microbiome diversity did on multifunctionality. However, only the underground soil microbiome diversity (locally abundant nematode richness, p = 0.002 and regionally abundant fungi richness, p = 0.048) had significantly effects on functional trade-off intensity (Figure.8c), the effect of aboveground plant species diversity (plant species richness, p = 0.063) was just 1/5 of that of underground soil microbial diversity.
