# Supplementary figures and images for: Shrub expansion raises both aboveground and underground multifunctionality on a subtropical plateau grassland: coupling multitrophic community assembly to multifunctionality and functional trade-off
Source: Front Microbiol. 2024 Jan 11;14:1339125. doi: 10.3389/fmicb.2023.1339125 (PMC10808678; doi:10.3389/fmicb.2023.1339125)

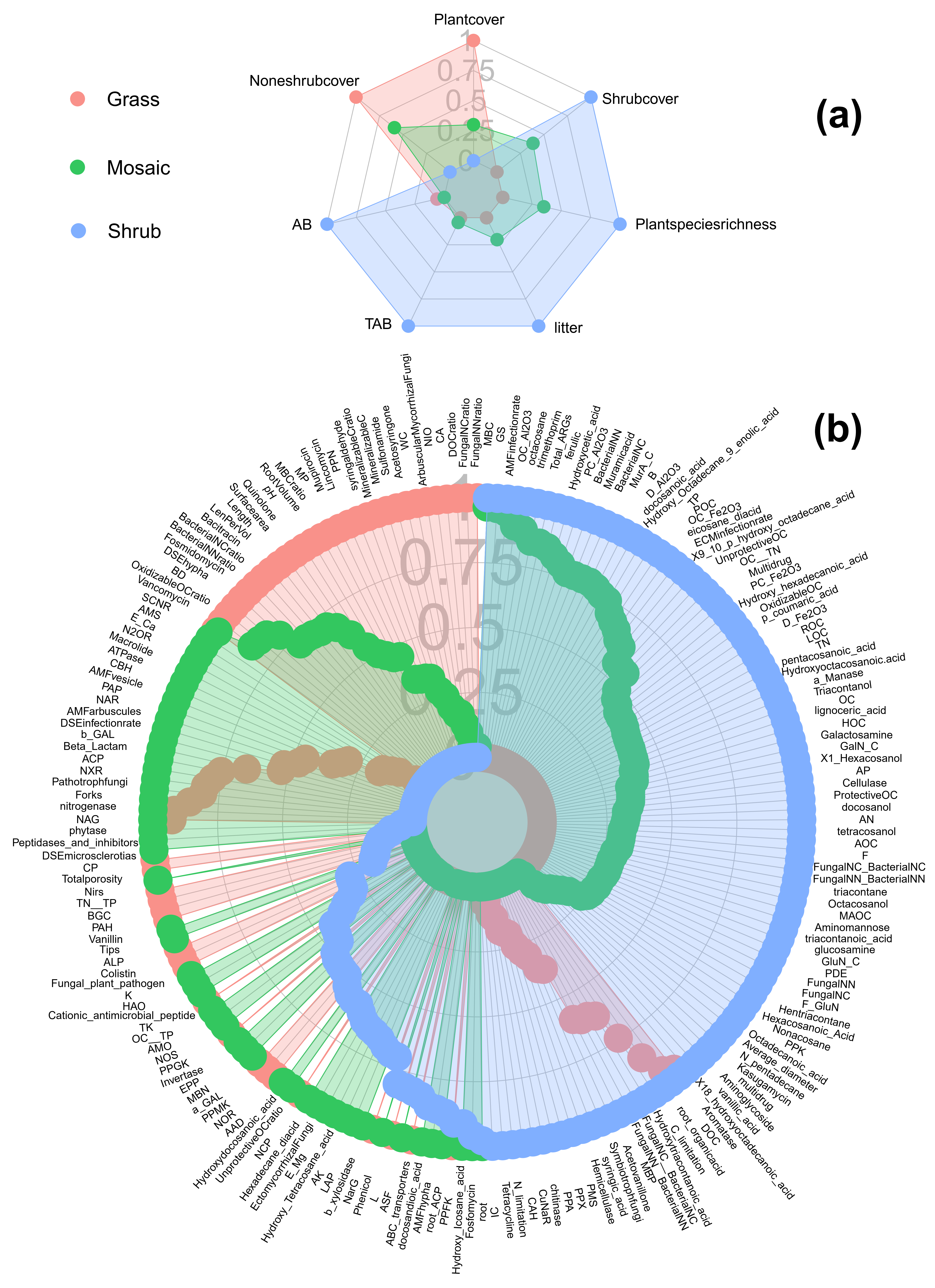

Supplement: Supplementary file 1 [file Data_Sheet_1.zip › Figure.S1.tif]

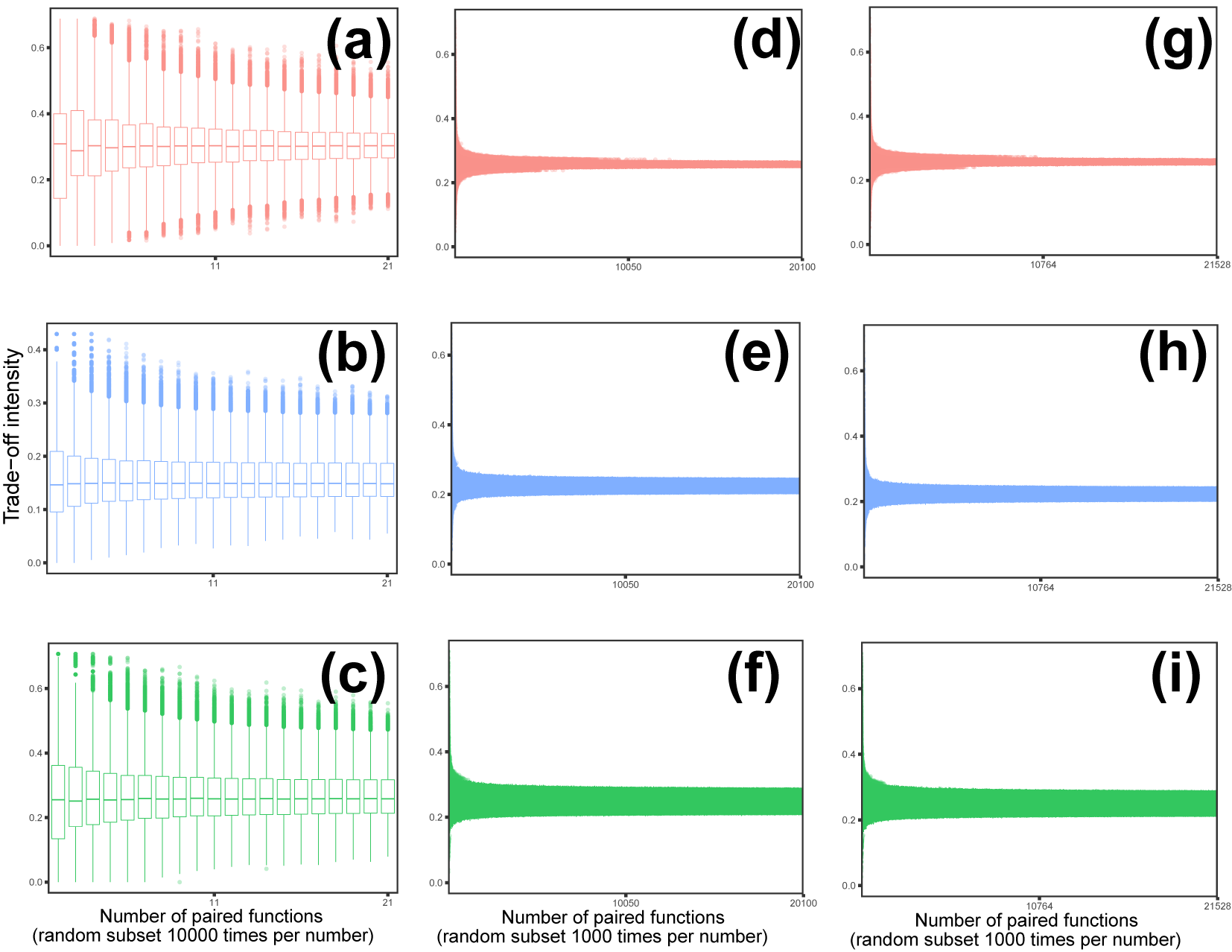

Supplement: Supplementary file 1 [file Data_Sheet_1.zip › Figure.S2.tif]

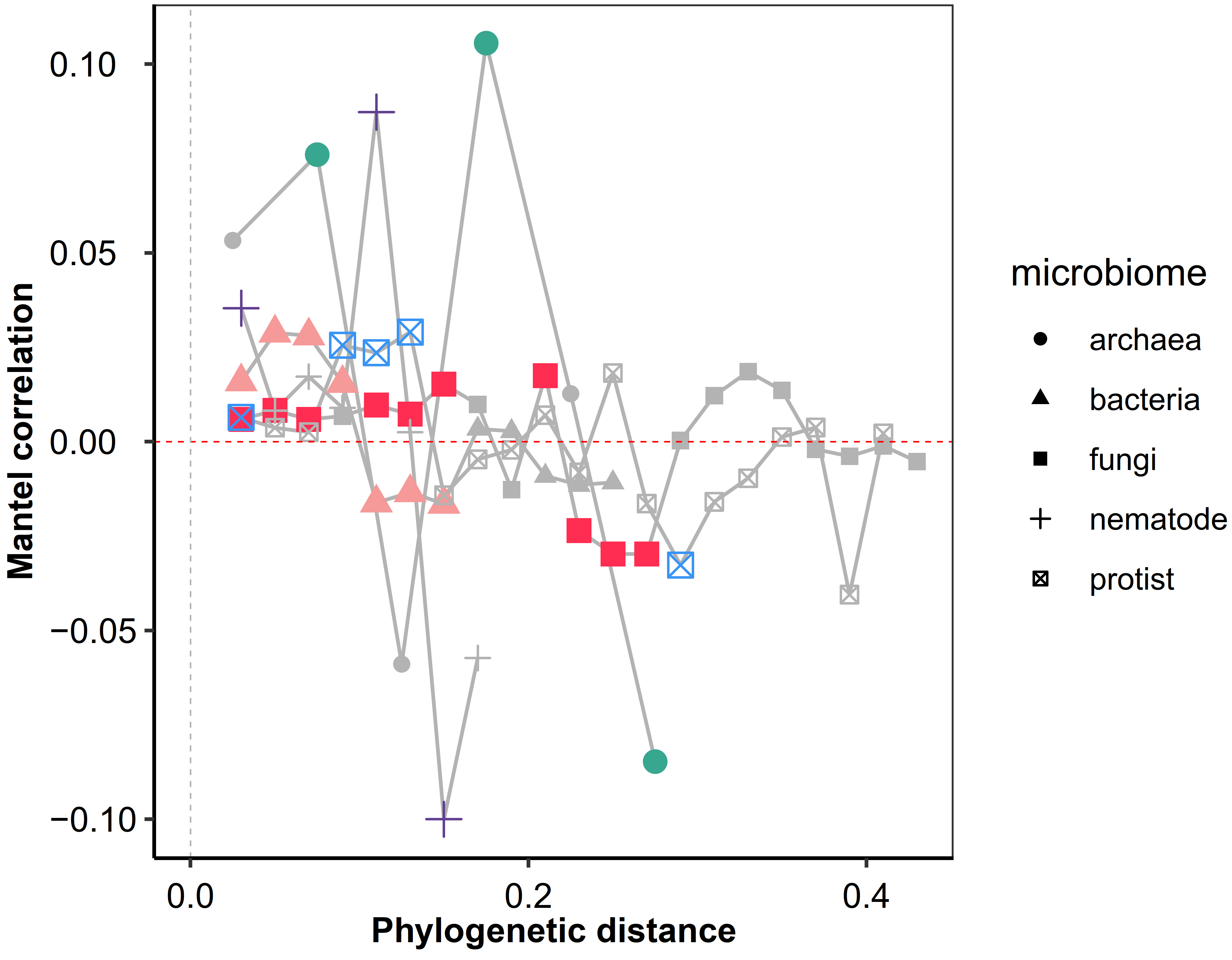

Supplement: Supplementary file 1 [file Data_Sheet_1.zip › Figure.S3.tif]

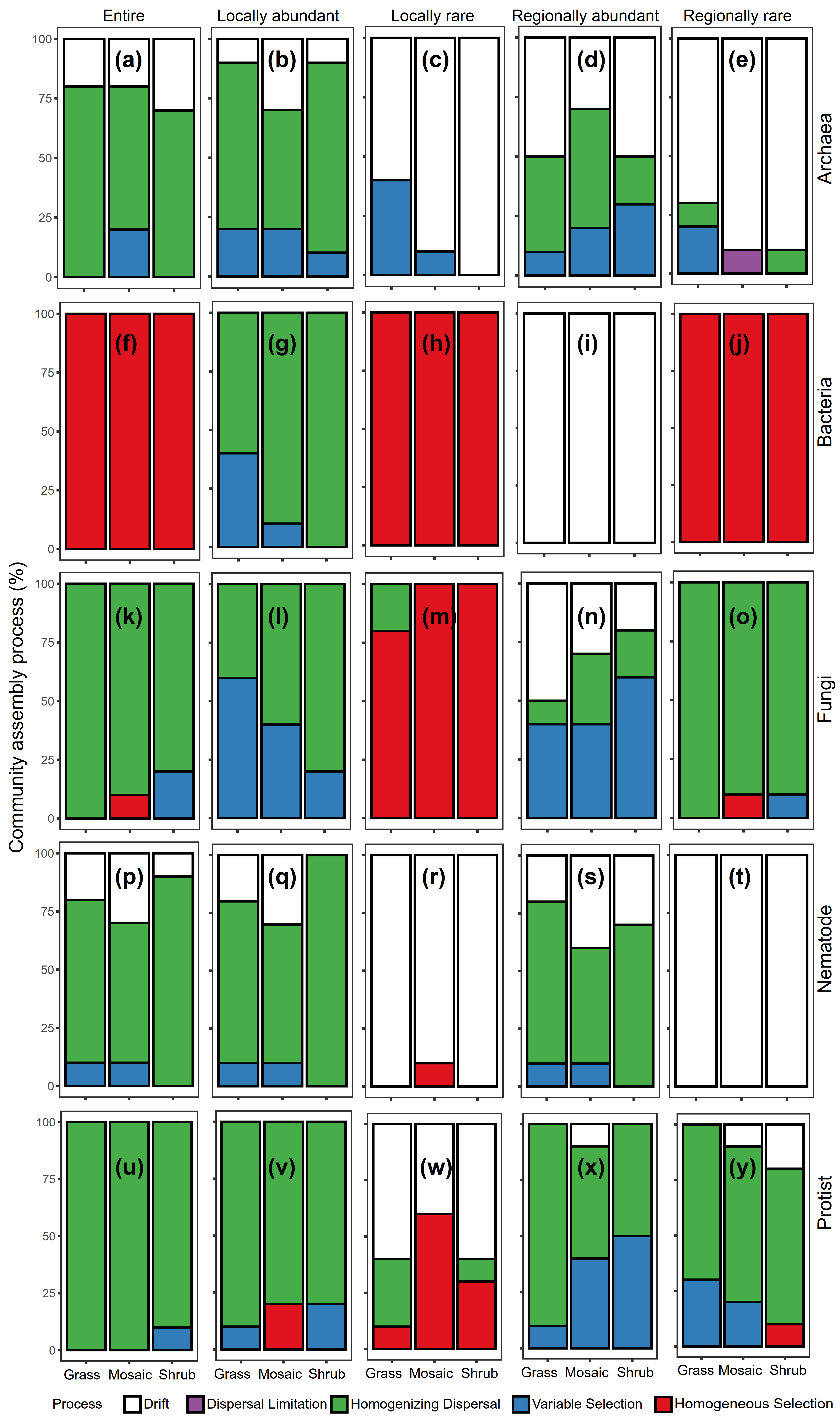

Supplement: Supplementary file 1 [file Data_Sheet_1.zip › Figure.S4.tif]

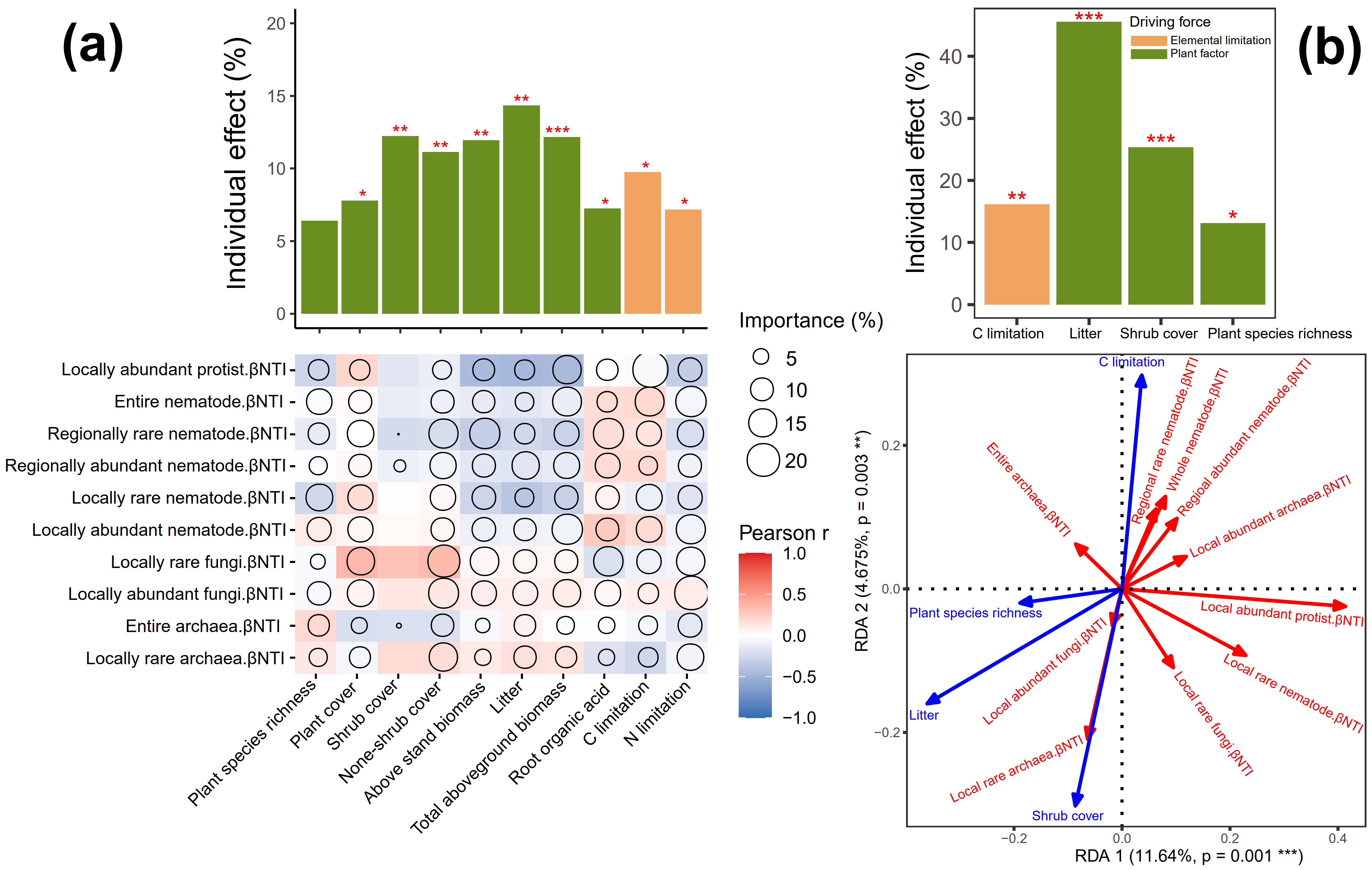

Supplement: Supplementary file 1 [file Data_Sheet_1.zip › Figure.S5.tif]

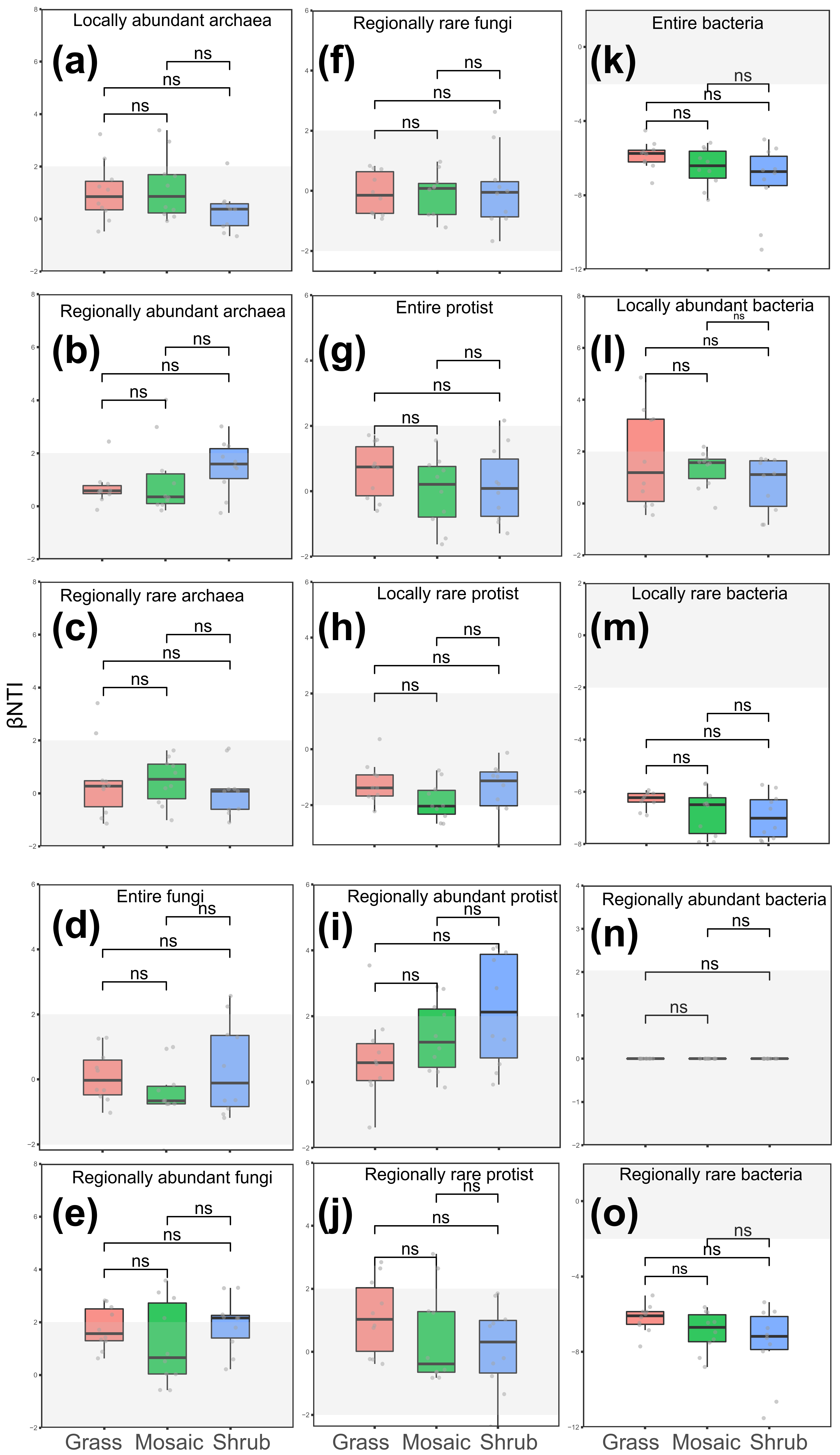

Supplement: Supplementary file 1 [file Data_Sheet_1.zip › Figure.S6.tif]

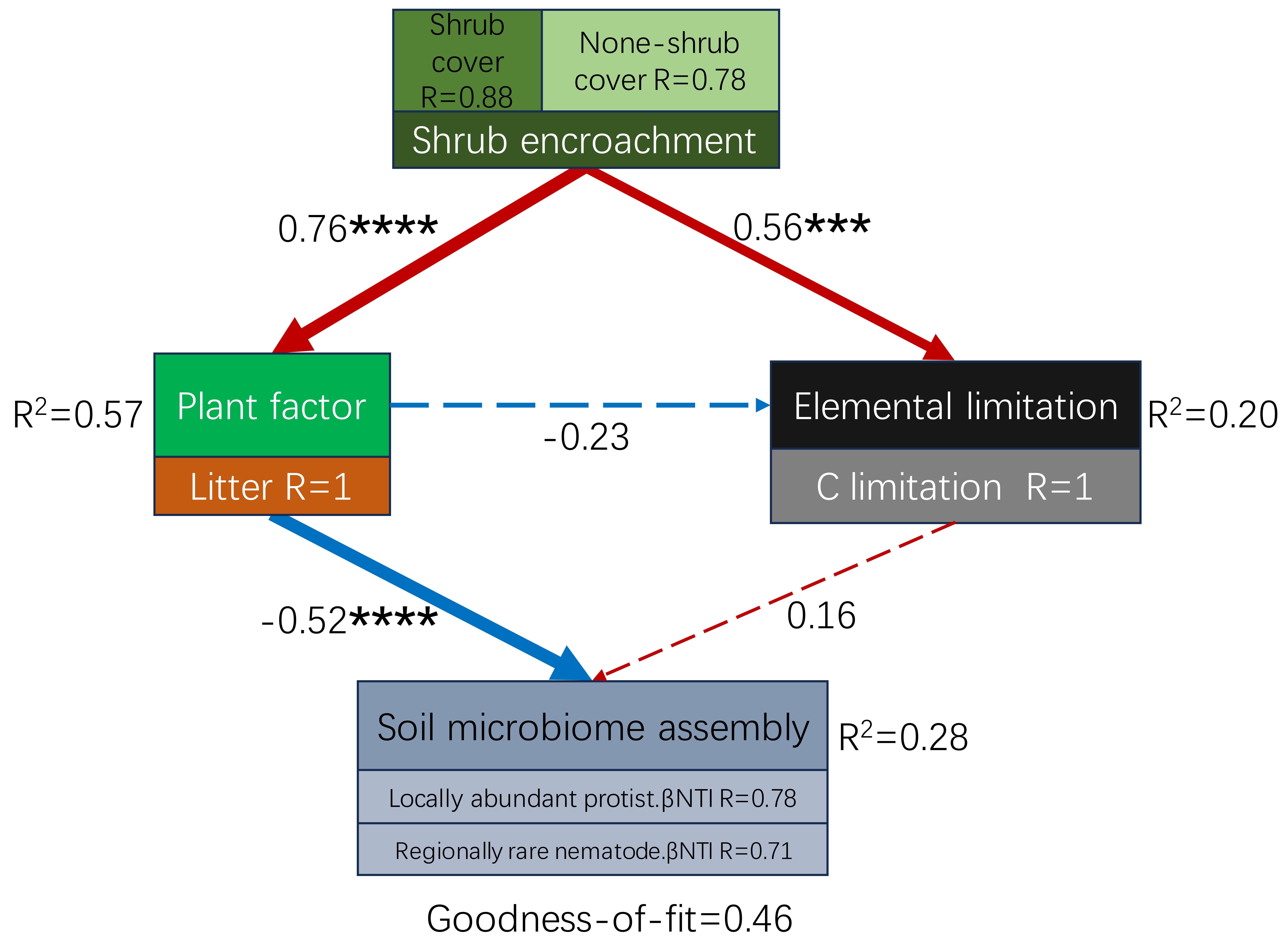

Supplement: Supplementary file 1 [file Data_Sheet_1.zip › Figure.S7.tif]

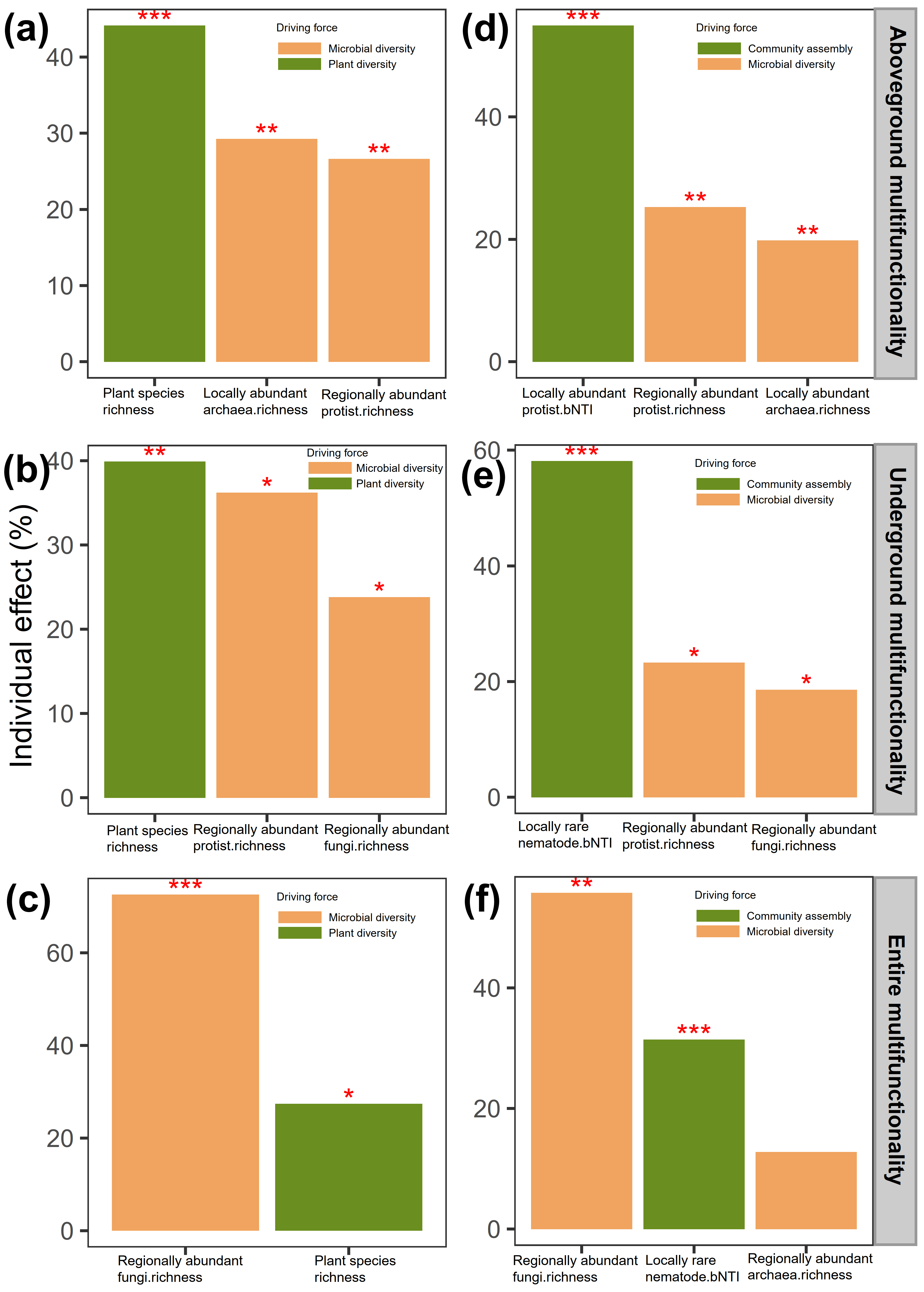

Supplement: Supplementary file 1 [file Data_Sheet_1.zip › Figure.S8.tif]
